# Diagnostic performance benchmarks of MRI for prostate cancer: a large-scale cohort study of 33,759 patients

**DOI:** 10.1186/s13244-026-02460-9

**Published:** 2026-11-04

**Authors:** Jie Bao, Mulin Su, Huijuan Tu, Litao Zhao, Yanting Ji, Pengfei Jin, Libiao Ji, Yueting Su, Hailin Shen, Ximing Wang, Xiaoying Wang, Zhen Li, Chunhong Hu, Yudong Zhang

**Affiliations:** 1 Department of Radiology, The First Affiliated Hospital of Soochow University, Suzhou, China; 2 Institute of Medical Imaging, Soochow University, Suzhou, China; 3 Medical Imaging, Soochow University, Suzhou, China; 4 Department of Radiology, Kunshan Hospital of Chinese Medicine, Kunshan, China; 5 Department of Computer Science and Engineering, The Chinese University of Hong Kong, Shatin, New Territories, Hong Kong, China; 6 Department of Radiology, Zhangjiagang People’s Hospital, Suzhou, China; 7 Department of Radiology, Zhejiang Cancer Hospital, Hangzhou, China; 8 Department of Radiology, Changshu No.1 Hospital, Changshu, China; 9 Department of Radiology, The People’s Hospital of Taizhou, Taizhou, China; 10 Department of Radiology, Suzhou Kowloon Hospital, Suzhou, China; 11 Department of Radiology, Peking University First Hospital, Beijing, China; 12 Department of Radiology, Tongji Hospital Tongji Medical College of HUST, Wuhan, China; 13 Department of Radiology, The First Affiliated Hospital of Nanjing Medical University, Nanjing, China

**Keywords:** Prostatic neoplasms, Magnetic resonance imaging, Multiparametric magnetic resonance imaging, Multicenter studies as topic

## Abstract

**Objective:**

To evaluate the real-world diagnostic performance of PI-RADS for detecting clinically significant prostate cancer (csPCa) across large-scale worldwide cohorts.

**Materials and methods:**

This multicenter observational study included 20,429 patients from nine Chinese institutions and a published worldwide meta-analysis dataset comprising 13,330 patients for comparison. All PI-RADS assessments were extracted from original MRI reports produced by clinical radiologists as part of standard diagnostic workflows. Summary receiver operating characteristic curves were constructed using a bivariate random-effects model, and center-level areas under the curve (AUCs) were pooled via random-effects meta-analysis. Subgroup analyses were conducted.

**Results:**

PI-RADS exhibited overall discriminatory performance for csPCa across nine Chinese cohorts comparable to the META results from 70 studies, though significant inter-center variation was observed. Using a PI-RADS ≥ 3 threshold, pooled sensitivity was 92.6% and specificity was 51.5%. At a PI-RADS ≥ 4 threshold, pooled specificity improved to 78.1%, with a sensitivity of 81.5%. The overall diagnostic performance was consistent across thresholds: The summary ROC (SROC) AUC was 0.903 for PI-RADS ≥ 3 and 0.826 for PI-RADS ≥ 4 in detecting csPCa at the meta-analysis level. Subgroup analyses indicated better performance among older patients (≥ 70 years) and those with higher PSA levels (> 20 ng/mL). PI-RADS v2.1 outperformed v2 or earlier versions in most clinical scenarios.

**Conclusions:**

This large-scale real-world study provides critical multicenter evidence supporting the use of PI-RADS in clinical practice worldwide, while also revealing substantial variability in scoring and diagnostic performance across institutions. The findings offer an evidence-based overview of PI-RADS implementation in China and worldwide, highlighting both its utility and inconsistencies.

**Key Points:**

***Question*** What are the real-world PI-RADS diagnostic performance benchmarks for clinically significant prostate cancer detection across diverse Chinese multicenter institutions versus global data?

***Findings*** PI-RADS demonstrated good overall performance (SROC AUC 0.903 at a threshold of ≥ 3), with superior discriminatory ability in older patients, higher PSA levels, and v2.1 users.

***Critical relevance statement*** This article critically assesses substantial inter-institutional heterogeneity in PI-RADS performance, highlighting that pooled estimates should not replace local calibration and that regionally adapted implementation strategies are essential for advancing clinical radiology practice.

**Graphical Abstract:**

**Figure:**
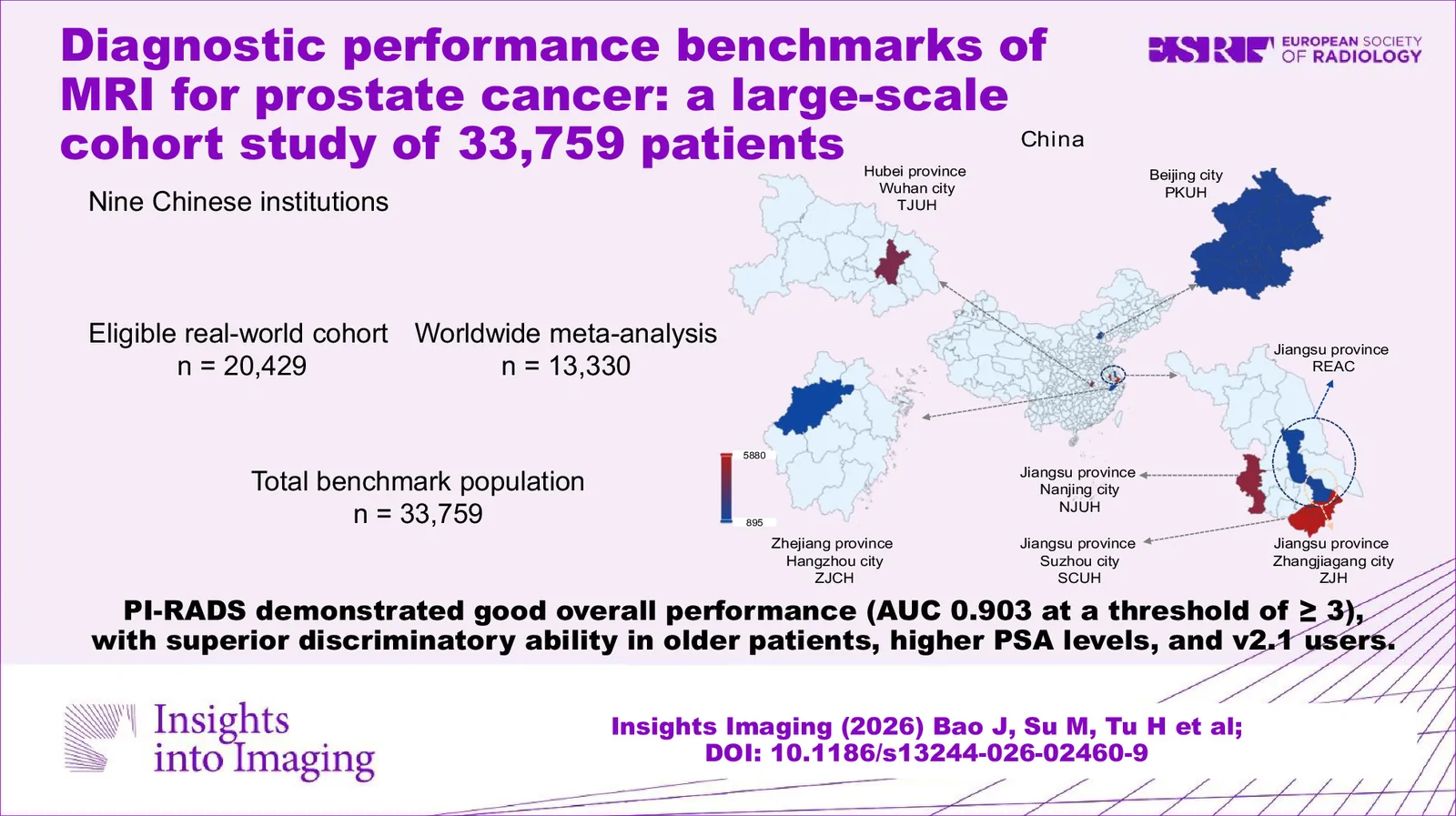

## Introduction

Prostate cancer (PCa) is a genomically heterogeneous disease with highly variable clinical outcomes [1, 2]. Previous studies have shown that clinically insignificant, indolent PCa is associated with lower cancer-specific mortality, whereas clinically significant PCa (csPCa) often progresses to advanced-stage disease [1, 3].

Current European Association of Urology (EAU) guidelines recommend magnetic resonance imaging (MRI) before prostate biopsy in all men with clinical suspicion of prostate cancer, reflecting the evidence that pre-biopsy MRI improves cancer detection rates and reduces unnecessary biopsies [4–6]. Since the introduction of the Prostate Imaging-Reporting and Data System version 2 (PI-RADS v2) by the European Society of Urogenital Radiology (ESUR) in 2015 [7], and its subsequent update to version 2.1 (PI-RADS v2.1) in 2019 [8], this framework has been widely adopted internationally as the standard for reporting multiparametric MRI (mpMRI) in the detection and characterization of csPCa [7, 9].

With the rapid and broad adoption of PI-RADS, its diagnostic accuracy and cancer detection rates (CDRs) have been extensively studied. Oerther et al conducted a systematic review and meta-analysis of PI-RADS v2.1 for detecting csPCa across 70 studies involving 13,330 patients [10]. They reported a sensitivity of 96% and specificity of 43% using a PI-RADS score ≥ 3, and a sensitivity of 89% and specificity of 66% using a score ≥ 4. Another meta-analysis from 2021, which included 10 studies with 1248 patients, reported a sensitivity of 87% and specificity of 74% [11]. These variations in diagnostic performance may be influenced by several factors, including MRI equipment field strength, scanning protocols, radiologists’ experience, and the reference standard used for pathological biopsy. Importantly, ethnic variations, such as differences in prostate cancer biological behavior, incidence rates (e.g., the higher incidence-to-mortality ratio of PCa in China compared to developed Western countries), and mpMRI manifestations, may limit the generalization of Western-derived validation data to Chinese populations [12–14].

Compared to the rapid advancement of MRI technology, the standardization of prostate MRI reporting in China has progressed more slowly [15]. Although PI-RADS guidelines have been disseminated globally, their standardized reporting templates have not yet been widely implemented in China [16]. Disparities in MRI equipment, examination techniques, and diagnostic expertise across domestic medical institutions further hinder the optimal utilization of MRI’s diagnostic potential. Additionally, many hospitals in China and worldwide lack access to transrectal MRI-ultrasound fusion biopsy systems, meaning histopathological confirmation is not always targeted, potentially affecting the accurate evaluation of mpMRI efficacy [8, 17, 18]. Therefore, a well-designed, large-sample study validating the diagnostic performance of PI-RADS in the Chinese population is both valuable and urgent.

To address this critical need for region-specific validation, this study aims to evaluate the diagnostic performance of PI-RADS for csPCa in China and worldwide cohorts. We designed a large-scale, multicenter retrospective study enrolling patients from geographically and demographically diverse institutions across China, reflecting real-world clinical practice. By systematically correlating PI-RADS assessments from routine radiological reports with histopathological outcomes, we seek to quantify the accuracy and variability of PI-RADS across different MRI platforms, radiologists, and healthcare settings. The findings of this study are expected to provide high-quality evidence to support the standardization of prostate MRI interpretation and reporting in China. Ultimately, this work aims to improve the precision and equity of csPCa diagnosis and establish a robust evidence base for future research on ethnic-specific diagnostic refinements.

## Materials and methods

### Study cohort

**Fig. 1:**
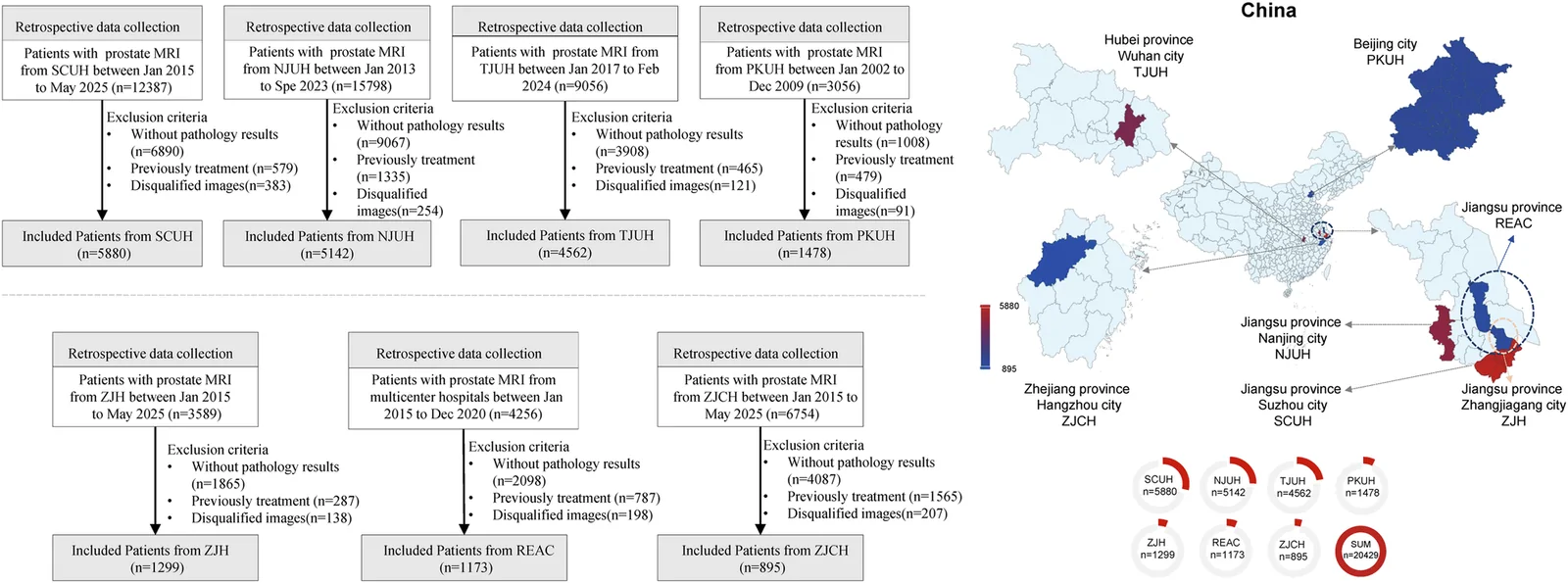
The flowchart of enrollment and geographic distribution of the multicenter study. The upper panel in geographic distribution depicts a map of China illustrating the geographic locations of the participating institutions. Magnified area insets provide detailed views of the provinces and cities where the institutions are located. The color of each highlighted region corresponds to the number of cases contributed by the institutions within that area, as indicated by the color scale on the left, ranging from blue (low, *n* = 895) to red (high, *n* = 5880). The lower panel consists of eight donut charts, each representing the number of participants at each institution and summary data. The length of the red arc is proportional to the value of the metric. The final chart represents the overall number of participants from all institutions. NJUH, the First Affiliated Hospital of Nanjing Medical University; SCUH, the First Affiliated Hospital of Soochow University; TJUH, Tongji Hospital, Tongji Medical College, Huazhong University of Science and Technology; PKUH, Peking University First Hospital; ZJCH, Zhejiang Cancer Hospital; REAC, a cross-district re-alliancing center (the REACs include the Changshu No.1 Hospital (CSH), Taizhou People’s Hospital (TZH), and Soochow Kowloon Hospital (SKH)); ZJH, Zhangjiagang People’s Hospital

This multicenter observational study retrospectively collected prostate imaging reports and corresponding clinical records between January 2002 and May 2025 from 5 tertiary medical centers, three cross-district re-alliancing centers (REACs), and a local county-level hospital. The five tertiary medical centers, including the First Affiliated Hospital of Nanjing Medical University (NJUH), the First Affiliated Hospital of Soochow University (SCUH), Tongji Hospital, Tongji Medical College, Huazhong University of Science and Technology (TJUH), Peking University First Hospital (PKUH), and Zhejiang Cancer Hospital (ZJCH), were classified as tertiary Grade A centers in China. The REACs, including the Changshu No.1 Hospital (CSH), Taizhou People’s Hospital (TZH), and Soochow Kowloon Hospital (SKH) in Jiangsu Province, were classified as district Grade B centers in China. Zhangjiagang People’s Hospital (ZJH) was classified as a county-level Grade C hospital in China. This retrospective, multicenter study was conducted in accordance with the ethical standards of the institutional research committees. The study protocol involving secondary analysis of anonymized data was reviewed and approved by the Institutional Review Boards (IRBs) of SCUH (Approval No. 2017-195) and NJUH (Approval No. 2024-SR-169), with a waiver of informed consent due to the retrospective nature of the study. The ethical approval was acknowledged or obtained from the review boards of all participating centers. The flowchart and geographic distribution of all participating institutions are illustrated in Fig. 1.

The inclusion criteria of the study were as follows: (1) men over 50 years of age with clinical suspicion of PCa (e.g., elevated PSA levels or abnormal digital rectal examination) who had undergone prostate MRI at 1.5 T or 3.0 T between January 2002 and May 2025; (2) patients with definitive histopathological results obtained via prostate biopsy or radical prostatectomy (RP). Biopsy decisions were made at the discretion of treating urologists based on a combination of clinical factors, including PI-RADS, PSA levels and kinetics, PSA density, digital rectal examination findings, and patient preference. Consequently, a proportion of biopsied patients had PI-RADS scores of 1–3, reflecting clinical scenarios in which biopsy was deemed warranted despite a low or intermediate MRI suspicion score, most commonly driven by elevated or rising PSA, high PSA density, or abnormal DRE findings independent of the MRI result. Patients were not systematically biopsied on the basis of PI-RADS score alone; (3) men with an initial negative biopsy were specially followed by PSA, DRE, MRI, and/or repeated TRUS biopsy with a follow-up period of at least 3 years. Men were excluded if they met the following criteria: (1) absence of histological examination or standard follow-up of at least 3 years; (2) absence of definitive structural imaging reports based on PI-RADS v2.1 or its earlier version; (3) a previous history of prostate biopsy or surgery or treatment for PCa (interventions for benign prostatic hyperplasia or bladder outflow obstruction were deemed acceptable). Finally, a total of 20,429 patients meeting the inclusion criteria were included from the participating institutions: SCUH (*n* = 5880), NJUH (*n* = 5142), TJUH (*n* = 4562), PKUH (*n* = 1478), ZJCH (*n* = 895), the cross-district institute group (REAC, *n* = 1173), and ZJH (*n* = 1299), respectively. In addition, a published dataset based on a worldwide meta-analysis [10] (META, *n* = worldwide meta-analysis 13,330) was obtained for comparison between China and the rest of the world.

### Histopathology and endpoint definitions

All histological specimens were evaluated by experienced pathologists in accordance with the 2014 International Society of Urological Pathology (ISUP) grading system. The primary outcome was the diagnostic accuracy of MRI for csPCa; the secondary outcome was its diagnostic accuracy for all PCa (csPCa + clinically insignificant PCa). csPCa was defined as ISUP Grade Group ≥ 2 (Gleason score ≥ 3 + 4 = 7). Clinically insignificant prostate cancer (ciPCa) was defined as ISUP Grade Group 1 (Gleason score 3 + 3 = 6). Detailed biopsy technique data, including approach (transrectal vs. transperineal), number of cores, and targeting method, were not available at the individual case level across all centers due to the retrospective design and heterogeneous clinical record systems spanning the study period. Based on institutional practice during the study period, the predominant approach was TRUS-guided systematic biopsy, with cognitive MRI-targeted biopsy increasingly adopted at tertiary centers from approximately 2015 onward. The details are shown in Supplementary Material 1.

### MRI acquisition and PI-RADS assessment

All MRI examinations were conducted using 1.5-T or 3.0-T scanners equipped with pelvic phased-array coils. The standardized multiparametric MRI protocol comprised T2-weighted imaging (T_2_WI) in axial, sagittal, and coronal planes, diffusion-weighted imaging (DWI) acquired with multiple b-values and corresponding apparent diffusion coefficient (ADC) maps, or dynamic contrast-enhanced (DCE) imaging following intravenous gadolinium-based contrast administration. Detailed institutional protocol parameters are provided in Supplementary Material (Table S1). PI-RADS assessment was performed through a structured dual-review process reflecting standard clinical workflow. Radiologists followed standardized training: 3-year residency with MRI training in years 3, followed by clinical practice without MRI reporting for 3 years, then prostate MRI reporting from year 3 onward with periodic prostate cancer MRI training. Prostate MRI examinations were initially evaluated by junior radiologists and subsequently reviewed by senior radiologists (> 10 years of genitourinary imaging experience). Discrepant scores were resolved through consensus discussion. Readers had access to clinical information (PSA, DRE, risk factors) during interpretation, consistent with routine practice. With the exception of PKUH (detailed below), all PI-RADS scores used in this study were extracted verbatim from the original clinical MRI reports generated at the time of each examination; no retrospective re-reading or re-scoring of these examinations was performed. For eight centers, PI-RADS scores therefore reflect guideline-concordant clinical practice at the time of reporting (PI-RADS v2 pre-2020; v2.1 from 2020 onward). For 1478 cases from PKUH (2002–2009, pre-PI-RADS era), images acquired prior to the introduction of the PI-RADS classification system, for which no PI-RADS scores existed in the original reports, were independently re-evaluated by two blinded radiologists using PI-RADS v2.1 criteria, with discrepancies resolved through consensus discussion. Among these PKUH cases, cancer-positive outcomes and a proportion of cancer-negative outcomes were confirmed histopathologically via biopsy or radical prostatectomy. For the remaining cancer-negative cases in whom biopsy was not performed, a negative reference standard was established based on structured clinical follow-up of 3–10 years comprising serial PSA measurement, digital rectal examination, and repeat MRI where clinically indicated, with no evidence of prostate cancer development throughout the follow-up period. This hybrid approach reflects real clinical practice while ensuring PI-RADS comparability across the historical PKUH cohort. Detailed reader qualifications and assessment strategies are provided in Supplementary Material 2.

### Statistical analysis

The diagnostic performance of PI-RADS classification for detecting PCa and csPCa was evaluated using two thresholds, i.e., score ≥ 3 and ≥ 4, respectively. Primary performance metrics, including cancer detection rate (CDR), sensitivity (SEN), specificity (SPE), positive predictive value (PPV), and negative predictive value (NPV), were calculated along with their 95% confidence intervals (CIs). Within each center, receiver operating characteristic (ROC) curves were analyzed, and area under the curve (AUC) values were estimated with 95% CIs using DeLong’s method. Summary ROC curves were constructed, and differences in AUCs were compared using the DeLong test. Pooled AUCs were derived using a random-effects model (restricted maximum likelihood, REML) following Fisher’s z transformation, with subsequent back-transformation to the AUC scale. Heterogeneity was assessed using *τ*², *I*², and the Cochran Q test.

Subgroup analyses were conducted to evaluate diagnostic performance based on the following categories: age (< 70 years vs. ≥ 70 years), PSA levels (< 10 ng/mL, 10–20 ng/mL, > 20 ng/mL), and MRI report release period (< 2020 vs. ≥ 2020; serving as a proxy for PI-RADS v2 vs. v2.1). Heterogeneity across studies was further examined using Higgins’ *I*² statistic, with *I*² > 50% indicating substantial heterogeneity.

Statistical analyses were carried out using MedCalc (v22.009, MedCalc Software), R (version 4.4.1) within the RStudio IDE (2025.05.1), and Python 3.11.7. A two-tailed *p*-value < 0.05 was considered statistically significant. To assess the robustness of the primary findings to the inclusion of pre-PI-RADS era cases, a sensitivity analysis was performed excluding the PKUH cohort (*n* = 1478; 7.2% of the total cohort). Additionally, diagnostic performance metrics (AUC, sensitivity, and specificity) were formally compared between PKUH and the non-PKUH cohort (*n* = 18,951) using DeLong’s method for AUC comparison and two-proportion *z*-tests for sensitivity and specificity. Results are presented in Supplementary Table S2.

## Results

### Baseline characteristics

In total, 20,429 patients from China were included in the primary analyses of diagnostic performance, compared with 13,330 patients from the worldwide META dataset. Overall, histological examination and follow-up using comprehensive clinical and imaging assessments yielded an overall csPCa rate of 46.6% (9527/20,429), with 51.2% (3011/5880), 50.9% (2618/5142), 44.5% (2029/4562), 23.7% (350/1478), 46.9% (610/1299), 33.7% (394/1173), and 57.5% (515/895) in SCUH, NJUH, TJUH, PKUH, ZJCH, REAC and ZJH, respectively, versus an overall rate of 33% (4399/13,330) in the META dataset. The baseline characteristics of patients included are described in Tables 1 and S3.

**Table 1:** The baseline characteristics of multicenter patients

Variable	SCUH, *n* = 5880			NJUH, *n* = 5142			TJUH, *n* = 4562			PKUH, *n* = 1478			ZJH, *n* = 1299			REAC, *n* = 1173			ZJCH, *n* = 895		
	PCa	Benign	*p*-value	PCa	Benign	*p*-value	PCa	Benign	*p*-value	PCa	Benign	*p*-value	PCa	Benign	*p*-value	PCa	Benign	*p*-value	PCa	Benign	*p*-value
No. of subjects	3258	2622		2923	2219		2229	2333		432	1046		696	603		486	687		582	313	
Age (years), median (IRQ)	72.0 (67.0, 76.0)	67.0 (61.2, 72.0)	≤ 0.0001	71.0 (65.0, 76.0)	66.0 (60.0, 71.0)	≤ 0.0001	73.0 (68.0, 79.0)	70.0 (64.0, 75.0)	≤ 0.0001	72.0 (67.0, 76.0)	70.0 (65.0, 75.0)		73.0 (69.0, 78.0)	70.0 (65.0, 75.0)	≤ 0.0001	73.0 (67.0, 77.0)	69.0 (64.0, 74.0)	≤ 0.0001	70.0 (65.0, 75.0)	67.0 (61.0, 72.0)	≤ 0.0001
Prostate zone, *n*	c	2622					2229	2333					696	603							
PI-RADS	3258	2662		2923	2219		2229	2333		432	1046		696	603		486	687		582	313	
PI-RADS 1–2	245 (7.5%)	976 (37.2%)	≤ 0.0001	201 (6.9%)	1058 (47.7%)	≤ 0.0001	277 (12.4%)	1400 (60.0%)	≤ 0.0001	45 (10.4%)	771 (73.7%)	≤ 0.0001	70 (10.1%)	282 (46.8%)	≤ 0.0001	36 (7.4%)	399 (58.1%)	≤ 0.0001	91 (15.6%)	180 (57.5%)	≤ 0.0001
PI-RADS 3	411 (12.6%)	774 (29.5%)	≤ 0.0001	273 (9.3%)	690 (31.1%)	≤ 0.0001	268 (12.0%)	590 (25.3%)	≤ 0.0001	42 (9.7%)	108 (10.3%)	0.799	109 (15.7%)	208 (34.5%)	≤ 0.0001	97 (20%)	222 (32.3%)	≤ 0.0001	57 (9.8%)	67 (21.4%)	≤ 0.0001
PI-RADS 4	831 (25.5%)	551 (21.0%)	≤ 0.0001	995 (34%)	393 (17.7%)	≤ 0.0001	396 (17.8%)	187 (8.0%)	≤ 0.0001	101 (23.4%)	121 (11.6%)	≤ 0.0001	113 (16.2%)	75 (12.4%)	0.0627	136 (28%)	55 (8.0%)	≤ 0.0001	155 (26.6%)	37 (11.8%)	≤ 0.0001
PI-RADS 5	1771 (54.4%)	321 (12.2%)	≤ 0.0001	1455 (49.7%)	78 (3.5%)	≤ 0.0001	1288 (57.8%)	156 (6.7%)	≤ 0.0001	244 (56.5%)	46 (4.4%)	≤ 0.0001	404 (58.0%)	38 (6.3%)	≤ 0.0001	217 (44.6%)	11 (1.6%)	≤ 0.0001	279 (47.9%)	29 (9.3%)	≤ 0.0001

Unless indicated otherwise, data are numbers of patients with percentage in parentheses. *p*-value was evaluated by a two-tailed *t*-test with unequal variance

*PCa* prostate cancer, *PZ* peripheral zone, *TZ* transition zone, *CZ* center zone, *AFMS* anterior fibromuscular stroma, *NJUH* the First Affiliated Hospital of Nanjing Medical University, *SCUH* the First Affiliated Hospital of Soochow University, *TJUH* Tongji Hospital, Tongji Medical College, Huazhong University of Science and Technology, *PKUH* Peking University First Hospital, *ZJCH* Zhejiang Cancer Hospital, *REAC* a cross-district re-alliancing center, *ZJH* Zhangjiagang People’s Hospital

**Fig. 2:**
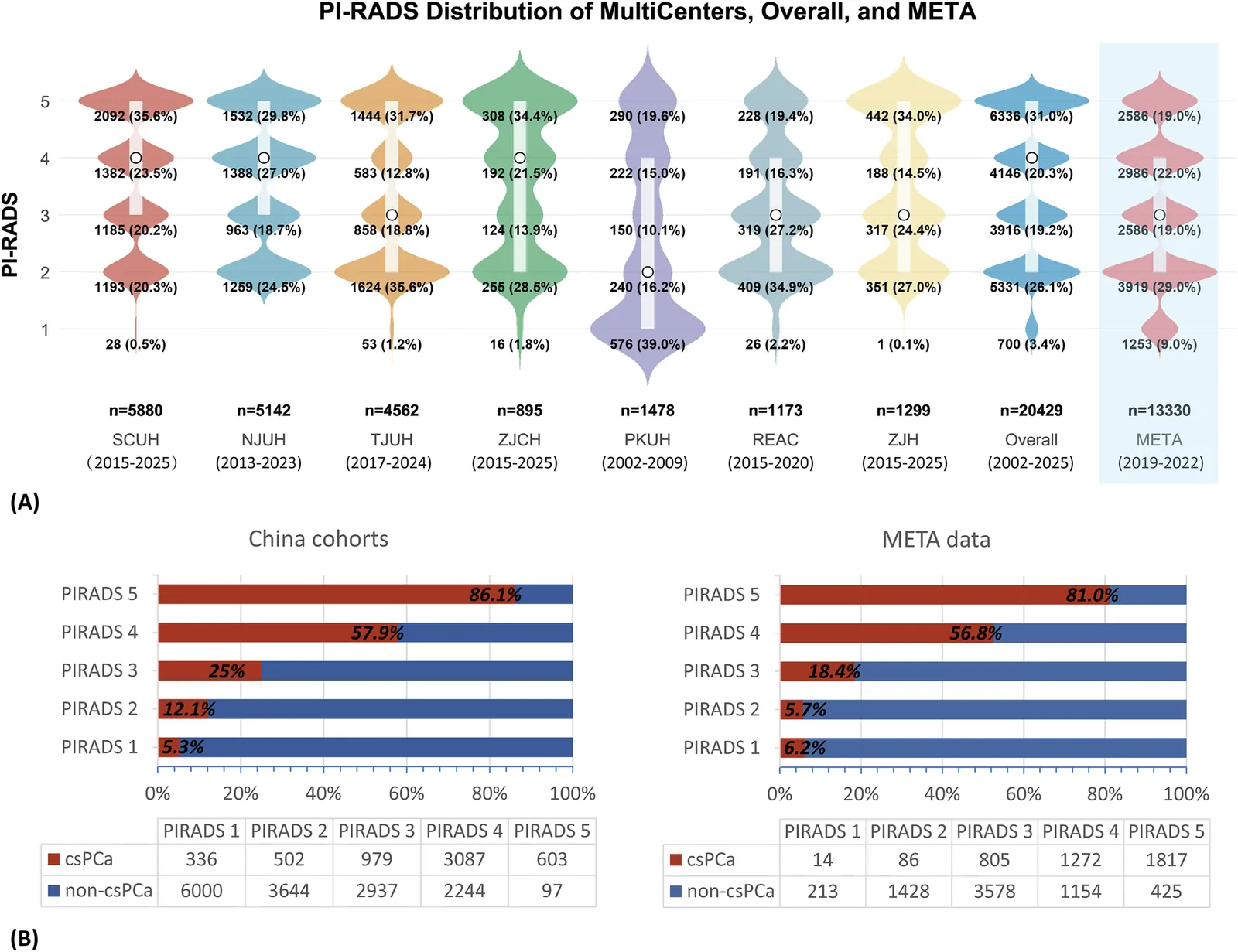
The violin plot of PI-RADS distribution of the multicenter cohort, overall cohort, and META dataset. **A** Each colored violin represents a single institution, with the final two plots displaying the aggregated data from the entire cohort and META analysis. The *y*-axis denotes the PI-RADS assessment categories, ranging from 1 to 5. The width of each violin is proportional to the density of cases at a given PI-RADS, providing a visual representation of the frequency distribution. Within each violin, the white circle indicates the median score, and the thicker vertical bar represents the interquartile range. The annotated numbers and percentages specify the absolute count and frequency of cases for each PI-RADS category within that center’s population. **B** Bar plots show the numbers and corresponding percentages of clinically significant prostate cancer (csPCa) vs. non-csPCa in individual PI-RADS categories

Specifically, the distribution of PI-RADS categories at each participating institution is summarized in Fig. 2. At the patient level (20,429 patients), the overall distribution of PI-RADS categories was as follows: 3.4% for PI-RADS 1, 26.1% for PI-RADS 2, 19.2% for PI-RADS 3, 20.3% for PI-RADS 4, and 31.0% for PI-RADS 5. In contrast, the META cohort at the patient level showed a slightly different distribution: 9.0% for PI-RADS 1, 29.0% for PI-RADS 2, 19.0% for PI-RADS 3, 22.0% for PI-RADS 4, and 19.0% for PI-RADS 5. The center-level analysis is presented in Supplementary Material 3.

### CDRs for PI-RADS category

**Fig. 3:**
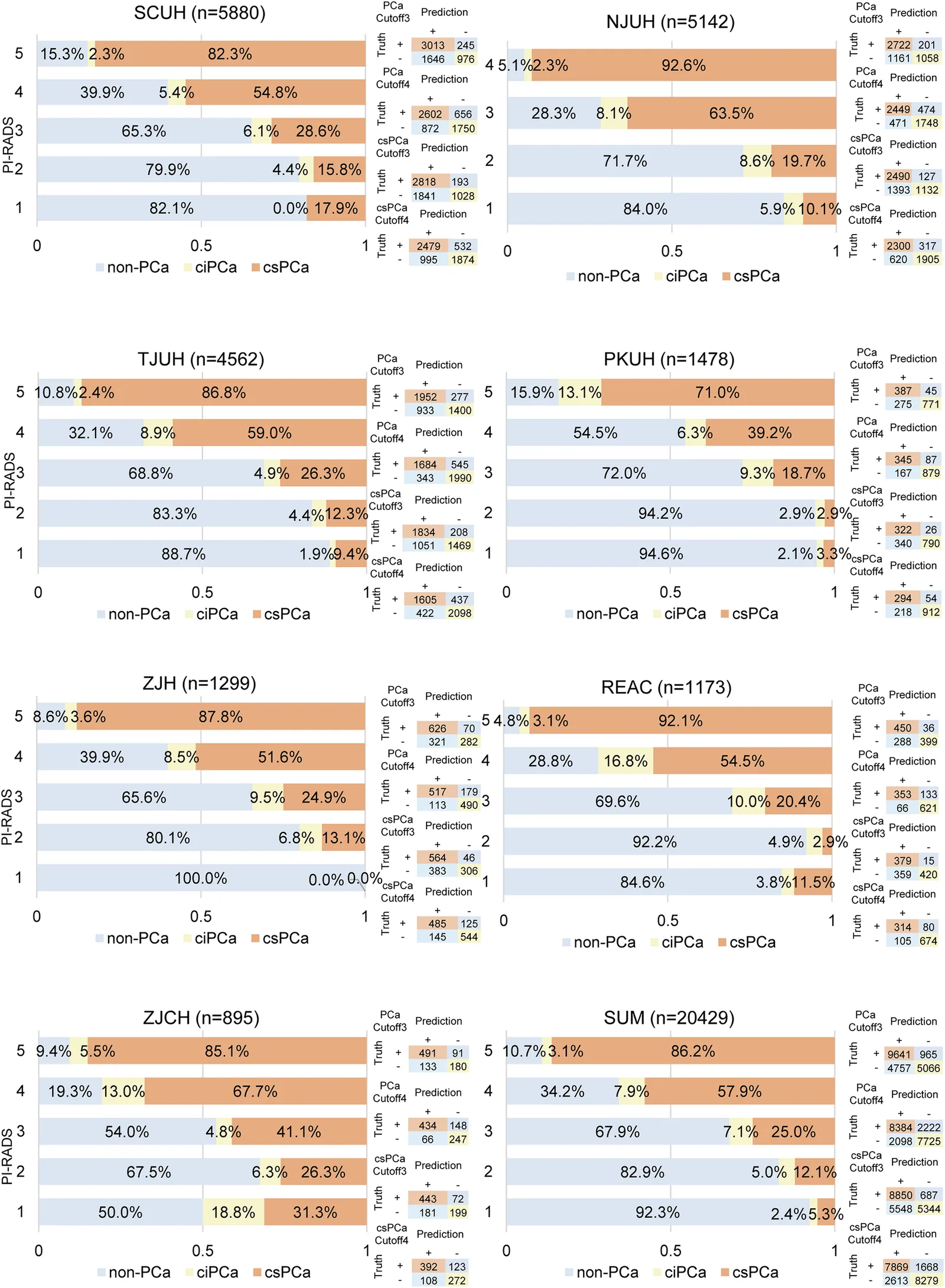
Distribution (non-PCa, ciPCa, and csPCa) and diagnostic performance of PI-RADS scores across the multicenter institutions. The figure is composed of eight panels: one for each of the seven participating centers and one for the aggregated sum of the overall participants (SUM). Within each panel, the stacked horizontal bar charts illustrate the distribution of histopathological outcomes (non-PCa, ciPCa, and csPCa) for each PI-RADS category (*y*-axis). The bars are segmented by color to represent non-prostate cancer (non-PCa, light blue), clinically insignificant prostate cancer (ciPCa, light orange), and clinically significant prostate cancer (csPCa, dark orange). The values annotated within each segment denote the absolute case count and the corresponding percentage within that specific PI-RADS category. To the right of the bar charts, confusion matrices summarize the diagnostic performance of PI-RADS. These matrices evaluate the ability of PI-RADS to detect any prostate cancer (PCa) and clinically significant prostate cancer (csPCa) at two different thresholds: PI-RADS of ≥ 3 and ≥ 4. Truth refers to the definitive histopathological diagnosis, while prediction refers to the diagnosis based on the PI-RADS cutoff

The proportions of benign findings, ciPCa, and csPCa for each PI-RADS category are illustrated in the horizontal bar charts in Fig. 3 (right panel). Overall, the CDR for csPCa increased with higher PI-RADS scores in both the Chinese and META cohorts: CDRs in the Chinese cohort were PI-RADS 1, 5.3%; PI-RADS 2, 12.1%; PI-RADS 3, 25.0%; PI-RADS 4, 57.9%; and PI-RADS 5, 86.2%. CDRs in the META dataset were as follows: PI-RADS 1, 6.2%; PI-RADS 2, 5.7%; PI-RADS 3, 18.4%; PI-RADS 4, 52.4%; and PI-RADS 5, 81%. Specifically, among the 20,429 patients in the Chinese cohorts, the rate of non-PCa, ciPCa, and csPCa in PI-RADS category 1 was 92.3%, 2.4%, and 5.3%, respectively. For PI-RADS category 2, the corresponding rates were 82.9%, 5.0%, and 12.1%. In patients with PI-RADS category 3, the rates were 67.9%, 7.1%, and 25.0% for non-PCa, ciPCa, and csPCa, respectively. For PI-RADS category 4, the rates were 34.2%, 7.9%, and 57.9% for non-PCa, ciPCa, and csPCa, respectively. Among those with PI-RADS category 5, the proportions were 10.7%, 3.1%, and 86.2% for non-PCa, ciPCa, and csPCa, respectively. Additionally, the PPV and NPV for csPCa at PI-RADS thresholds of ≥ 3 and ≥ 4 were calculated (left panel of Fig. 3). At score ≥ 3, the overall PPV and NPV for csPCa in Chinese cohorts were 61.5% (8850/14,398) and 88.6% (5344/6031), respectively, versus a PPV of 43.0% (3894/9051) and an NPV of 94.3% (1641/1741) in the META dataset. At score ≥ 4, the overall PPV and NPV for csPCa in Chinese cohorts were 75.1% (7869/10,482) and 83.2% (8279/9947), respectively, versus a PPV of 66.2% (3089/4668) and an NPV of 85.2% (5219/6124) in the META dataset. In addition, to illustrate the relationship between PI-RADS score and ISUP grade, the distribution of ISUP grade stratified by each PI-RADS score was plotted (Fig. S1).

### Diagnostic accuracy of PI-RADS

**Fig. 4:**
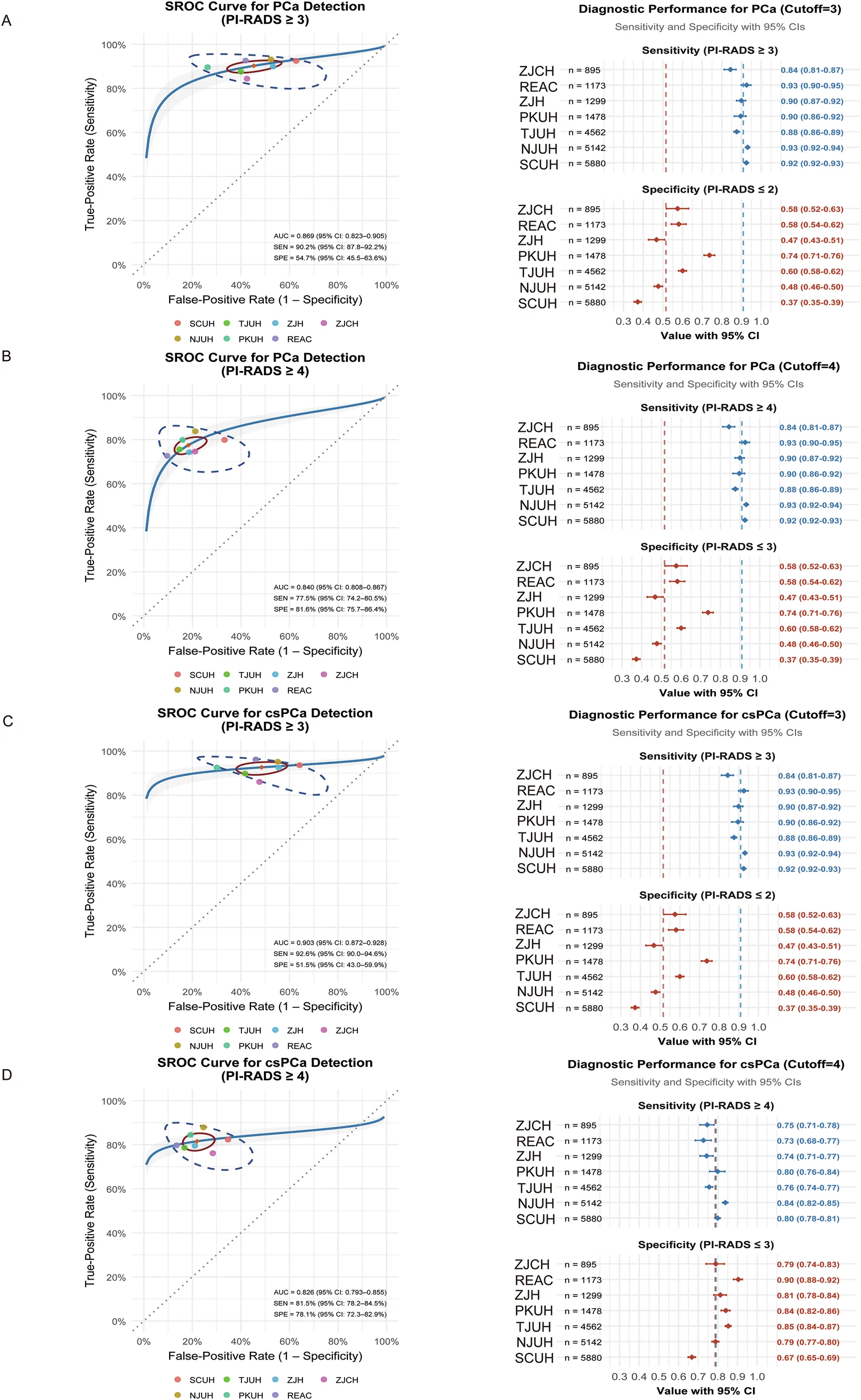
Summary ROC and coupled forest plots of the diagnostic performance of PI-RADS for detecting prostate cancer (PCa) and clinically significant prostate cancer (csPCa) at two different thresholds: PI-RADS of ≥ 3 and ≥ 4, respectively, in the multicenter institutions. The figure presents summary receiver operating characteristic (SROC) curves (left panels) and corresponding forest plots (right panels) for sensitivity and specificity. The analysis is stratified by two diagnostic targets: any PCa at PI-RADS thresholds of 3 (**A**) and 4 (**B**), and csPCa at PI-RADS thresholds of 3 (**C**) and 4 (**D**). On the SROC plots, each colored dot represents the sensitivity and specificity pair for an individual institution. The solid blue line is the summary ROC curve derived from a bivariate meta-regression model, with the gray shaded area indicating the 95% CI. The red ellipse represents the 95% CI for the summary operating point. The forest plots display the sensitivity (blue) and specificity (red) for each institution, with the horizontal bars representing the 95% CIs. AUC, area under the curve; SROC, summary receiver operating characteristic; SEN, sensitivity; SPE, specificity; CI, confidence interval

Considering potential inter-center variability for PI-RADS performance, a bivariate random-effects model was fitted to estimate the summary ROC curve across all China cohorts, from which the integrated AUC was derived. The summary ROC curves and corresponding forest plots for sensitivity and specificity, using both PI-RADS ≥ 3 and ≥ 4 as thresholds for detecting PCa and csPCa, are presented in Fig. 4 and Tables 2 and S4.

**Table 2:** The diagnostic performance of PI-RADS in predicting PCa and csPCa in multicenter

Centers		Cutoff	No. of patients	Positive	Negative	AUC	SEN	SPE
SCUH	PCa	≥ 3	5880	3258 (55.41%)	2622 (44.59%)	0.787 (0.776–0.797)	92.48 (91.5–93.4)	37.22 (35.4–39.1)
		≥ 4					79.86 (78.4–81.2)	66.74 (64.9–68.5)
	csPCa	≥ 3		3011 (51.21%)	2869 (48.79%)	0.795 (0.784–0.805)	93.59 (92.7–94.4)	35.83 (34.1–37.6)
		≥ 4					82.33 (80.9–83.7)	65.32 (63.5–67.1)
NJUH	PCa	≥ 3	5142	2923 (56.8%)	2219 (43.2%)	0.862 (0.853–0.872)	93.07 (92.1–94.0)	47.68 (45.6–49.8)
		≥ 4					83.74 (82.4–85.1)	78.72 (77.0–80.4)
	csPCa	≥ 3		2617 (50.9%)	2525 (49.1%)	0.872 (0.863–0.882)	95.08 (94.3–95.9)	44.84 (42.9–46.8)
		≥ 4					87.83 (86.6–89.1)	75.41 (73.7–77.1)
TJUH	PCa	≥ 3	4562	2229 (48.86%)	2333 (51.14%)	0.842 (0.831–0.853)	87.57 (86.1–88.9)	60.01 (58.0–62.0)
		≥ 4					75.55 (73.7–77.3)	85.30 (83.8–86.7)
	csPCa	≥ 3		2042 (44.76%)	2520 (55.24%)	0.852 (0.841–0.862)	89.81 (88.4–91.1)	58.29 (56.3–60.2)
		≥ 4					78.60 (76.8–80.4)	83.25 (81.7–84.7)
ZJH	PCa	≥ 3	1299	696 (53.58%)	603 (46.42%)	0.828 (0.806–0.848)	89.94 (87.5–92.1)	46.77 (42.7–50.8)
		≥ 4					74.28 (70.9–77.5)	81.26 (77.9–84.3)
	csPCa	≥ 3		610 (46.96%)	689 (53.04%)	0.844 (0.823–0.863)	92.46 (90.1–94.4)	44.41 (40.7–48.2)
		≥ 4					79.51 (76.1–82.6)	78.96 (75.7–81.9)
PKUH	PCa	≥ 3	1478	432 (29.23%)	1046 (70.77%)	0.878 (0.859–0.897)	89.23 (86.3–92.1)	73.62 (71.0–76.3)
		≥ 4					79.60 (75.8–83.4)	83.91 (81.7–86.1)
	csPCa	≥ 3		348 (23.5%)	1130 (76.5%)	0.875 (0.854–0.895)	92.06 (89.3–94.9)	69.84 (67.2–72.5)
		≥ 4					84.11 (80.3–87.9)	80.60 (78.3–82.9)
REAC	PCa	≥ 3	1173	486 (41.4%)	687 (58.6%)	0.876 (0.855–0.895)	92.6 (89.9–94.6)	58.1 (54.4–61.7)
		≥ 4					72.6 (68.5–76.4)	90.4 (88.0–92.4)
	csPCa	≥ 3	1173	394 (33.6%)	779 (66.4%)	0.895 (0.874–0.913)	96.2 (93.8–97.7)	53.9 (50.4–57.4)
		≥ 4					79.7 (75.4–83.4)	86.5 (83.9–88.7)
ZJCH	PCa	≥ 3	895	582 (65.03%)	313 (34.97%)	0.793 (0.765–0.819)	84.36 (81.2–87.2)	57.51 (51.8–63.1)
		≥ 4					74.57 (70.8–78.1)	78.91 (74.0–83.3)
	csPCa	≥ 3	895	515 (57.54%)	380 (42.46%)	0.777 (0.748–0.804)	86.02 (82.7–88.9)	52.37 (47.2–57.5)
		≥ 4					76.12 (72.2–79.7)	71.58 (66.8–76.1)

*PCa* prostate cancer, *csPCa* clinically significant prostate cancer, *NJUH* the First Affiliated Hospital of Nanjing Medical University, *SCUH* the First Affiliated Hospital of Soochow University, *TJUH* Tongji Hospital, Tongji Medical College, Huazhong University of Science and Technology, *PKUH* Peking University First Hospital, *ZJCH* Zhejiang Cancer Hospital, *ZJH* Zhangjiagang People’s Hospital, *REAC* a cross-district re-alliancing center

At a threshold of PI-RADS ≥ 3 at the patient level, the pooled sensitivity, specificity, and SROC AUC for detecting PCa were 90.2% (95% CI: 87.8–92.2%), 54.7% (95% CI: 45.5–63.6%), and 0.869 (95% CI: 0.823–0.905), respectively. For csPCa detection, the corresponding values were 92.6% (95% CI: 90.0–94.6%), 51.5% (95% CI: 43.0–59.9%), and 0.903 (95% CI: 0.872–0.928). At a threshold of PI-RADS ≥ 4 at the patient level, the pooled sensitivity, specificity, and SROCAUC for PCa detection were 77.5% (95% CI: 74.2–80.5%), 81.6% (95% CI: 75.7–86.4%), and 0.840 (95% CI: 0.808–0.867), respectively. For csPCa, these values were 81.5% (95% CI: 78.2–84.5%), 78.1% (95% CI: 72.3–82.9%), and 0.826 (95% CI: 0.793–0.855). Considerable heterogeneity was observed across multicenter data, with *I*² values for pooled sensitivity in detecting PCa and csPCa ranging from 93.0% to 93.6%, and for pooled specificity from 98.1% to 98.8%. These multicenter estimates mirror the META data (csPCa, patient level) that reported 96% sensitivity (95% CI: 95–98%) and 43% specificity (95% CI: 33–54%) for PI-RADS ≥ 3, and 89% sensitivity (95% CI: 85–92%) with 66% specificity (95% CI: 58–74%) for PI-RADS ≥ 4; corresponding AUCs were 0.86 and 0.89 [10]. Thus, across both our cohorts and META data, PI-RADS ≥ 3 maximizes cancer capture (90–96% sensitivity) while accepting considerable false-positive rates, whereas PI-RADS ≥ 4 sacrifices 5–10% sensitivity to gain 15–25% specificity. The SROC AUCs of both strategies cluster between 0.826 and 0.903, indicating that the choice of cutoff should be driven by clinical tolerance for missed cancers versus unnecessary biopsies rather than by any substantial difference in overall discriminative performance.

**Fig. 5:**
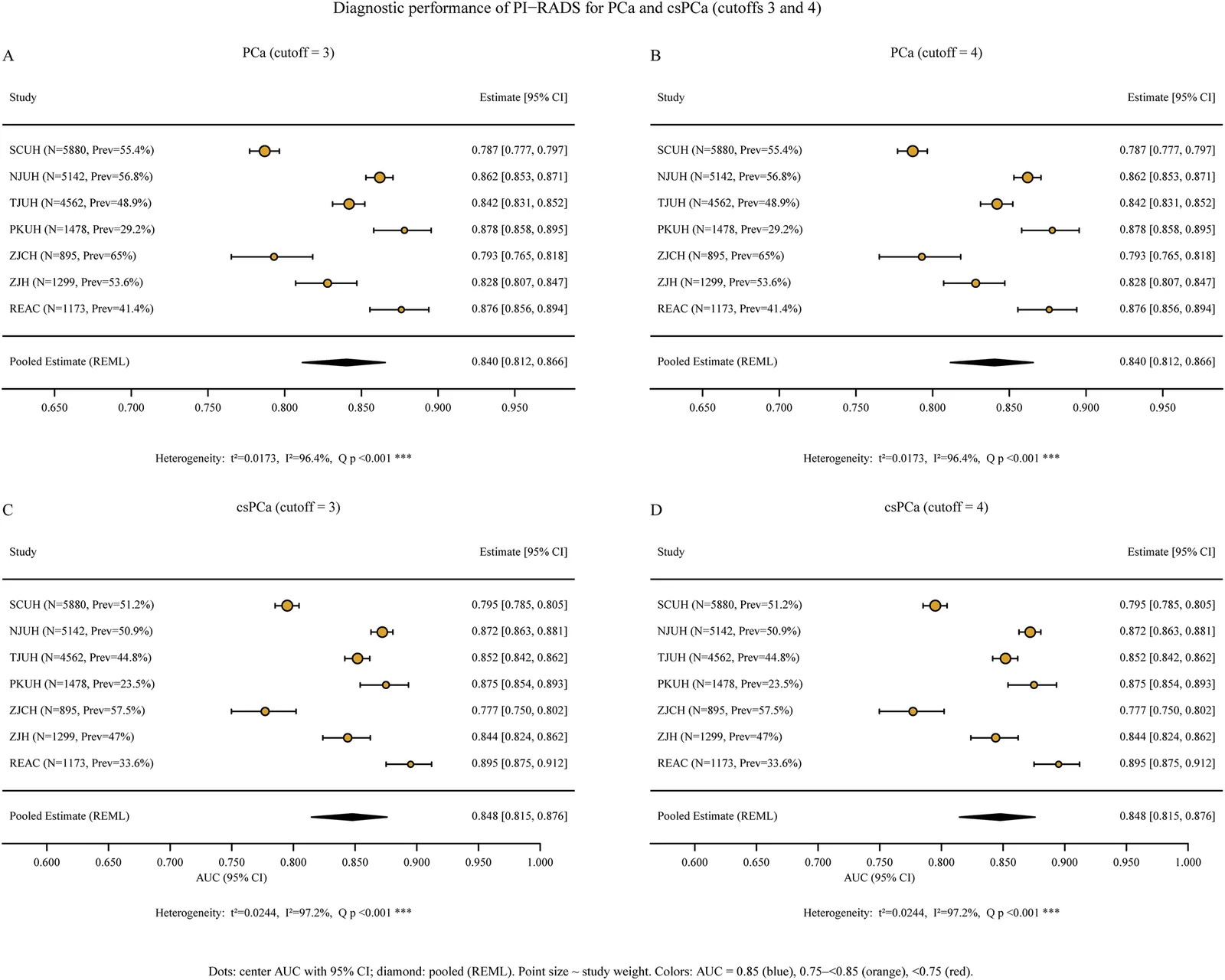
Forest plots summarizing the diagnostic performance of PI-RADS for prostate cancer (PCa; **A**, **B**) and clinically significant PCa (csPCa; **C**, **D**) at thresholds of ≥ 3 and ≥ 4. For each participating institution, yellow-filled circles show the AUC with 95% CI (DeLong). Marker size is proportional to the inverse-variance weight used in pooling. Diamonds indicate pooled AUCs obtained from random-effects meta-analysis (REML). Heterogeneity metrics (τ², I², Cochran Q *p*-value) are provided beneath each panel. Study labels display center identifier, total sample size (N), and disease prevalence

In parallel, a random-effects analysis was performed to pool center-specific AUCs in China cohorts. As shown in Fig. 5, PI-RADS demonstrated good discriminatory performance for both PCa and csPCa. The pooled AUCs for PCa detection were both 0.840 (95% CI: 0.812–0.866) at thresholds ≥ 3 and ≥ 4 (*I*² = 96.4%, *p* < 0.001). For csPCa, the pooled AUCs were 0.848 (95% CI: 0.815–0.876) at the same thresholds (*I*² = 97.2%, *p* < 0.001). These findings confirm substantial inter-site variability, likely driven by differences in MRI acquisition protocols, scanner hardware, radiologist experience, patient demographics, and disease prevalence. Pooled estimates should therefore be interpreted alongside this heterogeneity. To explore potential sources of heterogeneity and inform future standardization initiatives, we present subgroup analyses based on age, PSA levels, and PI-RADS versions in the following sections.

### Subgroup analysis

To further evaluate the diagnostic accuracy of PI-RADS for detecting PCa and csPCa, we conducted subgroup analyses based on patient age, PSA levels, and PI-RADS versions. Centers without available PSA data and PI-RADS version data because of clinical constraints were excluded from this subgroup analysis.

In the age-stratified analysis comparing patients < 70 years versus ≥ 70 years, significant differences in diagnostic performance were observed. Across most centers and thresholds, patients aged ≥ 70 years showed numerically higher AUCs compared with younger patients, and most differences were statistically significant (Fig. S2 and Table S5). Detailed results are provided in Supplementary Material 4.

In the PSA-stratified analysis (< 10 ng/mL, 10–20 ng/mL, and > 20 ng/mL) across five institutions and the summary cohort, AUC values generally increased with higher PSA levels for both PCa and csPCa detection (Fig. S3 and Table S6). Detailed results are provided in Supplementary Material 5. Overall, higher PSA levels were associated with better discrimination performance for both PCa and csPCa, whereas lower PSA levels correlated with reduced diagnostic accuracy, with some variation across institutions and thresholds.

Given that PI-RADS v2 was released in 2015 and v2.1 in 2019, we compared their diagnostic performance by grouping cases based on era of MRI examination (< 2020 vs. ≥ 2020). Detailed results are provided in Fig. S4, Table S7, and Supplementary Material 6. In summary, PI-RADS v2.1 generally demonstrated better diagnostic performance than v2 across most scenarios, although some center-specific variations were noted.

## Discussion

This diagnostic performance benchmark study comprising a large-scale cohort (*n* = 20,429) from nine medical centers across China and 13,330 patients from the worldwide META dataset suggests that PI-RADS exhibits overall discriminatory ability for detecting csPCa, though the extreme inter-center heterogeneity (*I*² = 93–99%) warrants caution in interpreting pooled estimates as generalizable performance benchmarks. Overall, there is no significant difference in the overall accuracy of PI-RADS between China and the rest of the world, though substantial inter-institutional heterogeneity in performance was observed. Notably, the distribution of PI-RADS categories varied significantly across centers, revealing distinct interpretive tendencies: some institutions displayed more aggressive scoring patterns (higher frequency of categories 4–5), while others adopted a more conservative approach (greater proportion of categories 1–2). Importantly, cancer risk increased monotonically with higher PI-RADS scores, where CDRs for csPCa rose consistently from approximately 5% in category 1 to 80% or more in category 5 in both the Chinese and META cohorts. This stepwise correlation strongly supports the predictive validity of PI-RADS in worldwide and Chinese clinical contexts.

To date, this multicenter study, with the largest dataset, reflects real-world outcomes of PI-RADS applied across highly heterogeneous medical centers in China for diagnosing csPCa. Encouragingly, despite significant inter-institutional variability, our performance results broadly align, as contextual benchmarks, with those reported in global meta-analyses, though direct numerical comparisons should be interpreted with caution given important methodological differences between datasets (detailed below). For instance, the systematic review and meta-analysis by Oerther et al [10], which included 70 studies, reported a summary sensitivity of 96% and specificity of 43% with an SROC AUC of 0.86 at PI-RADS ≥ 3, and 89% sensitivity and 66% specificity with an SROC AUC of 0.89 at PI-RADS ≥ 4. Similarly, Park et al [11] reported, across 10 eligible articles, a pooled sensitivity of 87% and specificity of 74% for diagnosing csPCa. It should be noted that comparisons between our cohort and these meta-analyses are intended as contextual benchmarking rather than direct equivalence testing, given the following key methodological differences: (1) Study design: our cohort retrospectively collected real-world PI-RADS reports from 9 Chinese institutions spanning tertiary, district, and county-level hospitals, whereas Oerther et al pooled 70 studies of mixed designs from multiple countries with heterogeneous reporting standards and MRI protocols. (2) Reference standard: our reference standard was histopathology graded per the 2014 ISUP classification, with a minimum 3-year clinical follow-up for patients with initial negative biopsies; the constituent studies of Oerther et al varied considerably in biopsy approach (targeted vs. systematic), follow-up duration, and csPCa threshold definition. (3) Disease prevalence: the csPCa prevalence in our cohort was 46.6% (9527/20,429), substantially higher than the 33% (4399/13,330) in the META data; such differences in case mix affect predictive values and may influence pooled sensitivity and specificity estimates. (4) PI-RADS category distribution: our cohort showed a higher proportion of PI-RADS 5 cases (31.0% vs. 19.0% in META) and fewer PI-RADS 1 cases (3.4% vs. 9.0%), reflecting differences in institutional referral patterns and reader behavior. (5) Heterogeneity: both datasets exhibited substantial internal heterogeneity (*I*² = 93.0–98.8% in our cohort), further limiting direct cross-dataset comparability. Despite these differences, the broad similarity in PI-RADS discriminative performance across our Chinese cohort and the worldwide meta-analytic estimates suggests that PI-RADS demonstrates diagnostic validity across distinct healthcare systems, MRI platforms, and patient populations, though the high inter-center heterogeneity underscores that performance should be interpreted within the local institutional context rather than as a fixed universal benchmark. Consistent with these studies, our findings demonstrate that the choice of threshold significantly influences diagnostic performance. At PI-RADS ≥ 3, it showed high pooled sensitivity but moderate specificity (PCa: 90.2% sensitivity, 54.7% specificity; csPCa: 92.6% sensitivity, 51.5% specificity). In contrast, at PI-RADS ≥ 4, sensitivity decreased while specificity improved (PCa: 77.5% sensitivity, 81.6% specificity; csPCa: 81.5% sensitivity, 78.1% specificity). Accordingly, the SROC AUC was higher at the ≥ 3 threshold (PCa: 0.869; csPCa: 0.903) than at ≥ 4 (PCa: 0.840; csPCa: 0.826), highlighting the differential operating points associated with each threshold. Notably, the pooled AUCs derived from a random-effects meta-analysis of center-level AUCs were similar across thresholds (PCa: 0.840; csPCa: 0.848), consistent with the fact that AUC based on continuous PI-RADS scores is a threshold-independent measure of discriminatory power. Therefore, these results suggest that PI-RADS offers overall discrimination between diseased and non-diseased cases across the studied institutions; however, given the extreme heterogeneity observed, individual center performance may deviate substantially from the pooled summary estimates. The selected clinical threshold governs the trade-off between sensitivity and specificity.

Second, substantial heterogeneity was observed across participating institutions. High *I*² values were identified in both the pooled AUC meta-analysis and the bivariate models. This variability likely stems from a combination of technical and clinical factors [8], including differences in MRI vendors, field strength, and sequence parameters; varying adherence to PI-RADS technical recommendations; disparities in radiologist training and experience; and center-specific case mix, including disease prevalence. Formal meta-regression to identify predictors of heterogeneity was not feasible due to the incomplete availability of center-level covariate data across all nine institutions. This limits our ability to attribute performance variation to specific technical or clinical factors. Notably, institutions with a higher proportion of PI-RADS 4–5 cases tended to exhibit greater specificity and PPV, whereas those with more conservative scoring practices demonstrated higher sensitivity. These effects, related to case spectrum and reader behavior, highlight the need for local calibration and robust quality assurance when implementing PI-RADS in clinical diagnostic pathways.

Subgroup analyses further support personalized threshold selection by aligning diagnostic performance with clinically actionable outcomes. Specifically, the higher diagnostic accuracy (reflected by increased AUCs) observed in older patients (≥ 70 years) and those with elevated PSA levels, particularly above 20 ng/mL, justifies the use of a PI-RADS ≥ 4 threshold in these high-risk subgroups. This strategy emphasizes specificity, effectively reducing unnecessary biopsies while preserving acceptable sensitivity. Conversely, in lower-risk populations (younger patients or those with PSA < 10 ng/mL), where avoiding missed diagnoses is critical, a PI-RADS ≥ 3 threshold is recommended to maximize sensitivity and reduce the risk of overlooking clinically significant PCa. These results advocate for a risk-stratified biopsy approach that deliberately balances the risks of overtreatment against those of underdiagnosis. The general superiority of PI-RADS v2.1 over v2 across most centers underscores the incremental refinements in the updated version. Isolated cases where PI-RADS v2 performed comparably or better likely reflect site-specific factors such as local imaging protocols, radiologists’ familiarity with the earlier version, or differences in patient characteristics, suggesting that the implementation of updated guidelines may be modulated by institutional context and experience. However, further research with larger sample sizes may be needed to confirm these findings and explore any potential subtle effects.

Our study has several limitations. First, although it adopted a retrospective multicenter design, the potential for selection and referral biases remains. Temporal variations in biopsy indications, MRI protocols, and radiologist experience could not be fully accounted for. Second, because of the retrospective nature of the study, not all men who underwent prostate MRI also underwent histopathological examination or full-cycle follow-up confirmation, possibly leading to underestimation or overestimation of the true disease prevalence. Formal statistical corrections (e.g., Begg-Greenes adjustment, multiple imputation) were not feasible given the absence of individual-level verification probability data across all centers. Readers should therefore interpret the reported diagnostic performance as reflective of real-world PI-RADS utilization patterns rather than accuracy under ideal conditions. Third, because PI-RADS scores were derived from routine clinical reports, reporting radiologists were not blinded to clinical information such as PSA levels, DRE findings, or prior biopsy results. This introduces the risk of incorporation bias, which may have inflated observed diagnostic performance relative to a blinded reading paradigm. However, this also means our results reflect actual clinical practice conditions, which enhances external validity. Fourth, no formal image quality assessment using structured tools such as PI-QUAL was performed. The retrospective design and the long study period precluded systematic prospective quality scoring. Finally, technical heterogeneity in MRI acquisitions, including differences in vendor, field strength, coil type, sequence selection, and adherence to PI-RADS technical recommendations, was present across institutions and not standardized. In particular, the 1478 cases from PKUH (7.2% of the total cohort) were acquired between July 2002 and December 2009, prior to the introduction of PI-RADS, and the original acquisition protocols may not have been fully compliant with current PI-RADS technical recommendations. To mitigate this limitation, these cases were retrospectively scored by two experienced radiologists using PI-RADS v2.1 criteria under blinded, dual-read conditions with consensus resolution of discrepancies. A formal sensitivity analysis excluding PKUH yielded results fully consistent with the primary findings (ΔAUC ≤ 0.007; Δ sensitivity ≤ 0.1%; Δ specificity ≤ 2.7%), and the observed differences in AUC and specificity between PKUH and the non-PKUH centers were attributable to PKUH’s substantially lower disease prevalence (csPCa: 23.5% vs. 48.5%) rather than to systematic bias from acquisition non-compliance. The primary conclusions of this study are therefore robust to the inclusion of these historically acquired cases.

## Conclusion

Based on a large-scale multicenter Chinese cohort compared with the worldwide META dataset, this study demonstrates that PI-RADS provides overall discrimination between PCa and csPCa across diverse real-world institutions, with higher scores correlating strongly with csPCa risk and performance broadly consistent with international benchmarks. While PI-RADS ≥ 3 maximizes sensitivity, PI-RADS ≥ 4 offers a more balanced performance. Significant inter-institutional variation was observed, attributable to differences in imaging protocols, interpretation thresholds, and patient characteristics, underscoring that PI-RADS performance is context-dependent rather than uniformly fixed. Given the substantial inter-institutional heterogeneity observed, pooled estimates should be interpreted as descriptive summaries of real-world practice rather than as universally generalizable performance standards. Subgroup analyses linked improved accuracy to older age and higher PSA levels. PI-RADS v2.1 generally outperformed v2, supporting its ongoing adoption. These real-world findings highlight the need for standardized implementation, robust quality assurance, and prospective validation to further consolidate the role of PI-RADS in prostate cancer diagnosis across China.

## Supplementary information

ELECTRONIC SUPPLEMENTARY MATERIAL

## Data Availability

Interested users may request access to these data, where institutional approvals, along with signed data use agreements and/or material transfer agreements, may be needed/negotiated. Derived result data supporting the findings of this study are available upon reasonable request.

## Abbreviations

AUC: Area under the curve
CDR: Cancer detection rate
CIs: Confidence intervals
csPCa: Clinically significant PCa
ISUP: International Society of Urological Pathology
NPV: Negative predictive value
PCa: Prostate cancer
PI-RADS v2: Prostate Imaging-Reporting and Data System version 2
PPV: Positive predictive value
REACs: Re-alliancing centers
ROC: Receiver operating characteristic
SEN: Sensitivity
SPE: Specificity

## Declarations

### Study subjects or cohorts overlap

Some study subjects or cohorts have been previously reported in the *British Journal of Cancer* and *European Journal of Nuclear Medicine and Molecular Imaging* by our team.
