# Supplementary material for: Diagnostic performance benchmarks of MRI for prostate cancer: a large-scale cohort study of 33,759 patients
Source: Insights Imaging. 2026 Nov 4;17:278. doi: 10.1186/s13244-026-02460-9 (PMC13737363; doi:10.1186/s13244-026-02460-9)
Supplement: Supplementary file 1 [file 13244_2026_2460_MOESM1_ESM.pdf]

Diagnostic Performance Benchmarks of MRI for Prostate Cancer: a Large-Scale Cohort Study of 33,759 Patients

ELECTRONIC SUPPLEMENTARY MATERIAL

Supplementary Material 1

For patients who underwent both biopsy and subsequent radical prostatectomy (RP), the RP specimen served as the definitive pathological reference. In cases where only biopsy was performed, the biopsy result was utilized. Histopathological findings were classified based on the ISUP Grade Group (GG) system as follows: benign (no carcinoma), ISUP GG 1 (Gleason Score, GS 3+3), ISUP GG 2 (GS 3+4), ISUP GG 3 (GS 4+3), ISUP GG 4 (GS 8), and ISUP GG 5 (GS 9-10). The csPCa was defined as ISUP GG ≥ 2, and clinically insignificant PCa (ciPCa) was defined as ISUP GG 1. In addition, patients without histopathologic results were grouped as PCa if they had consistently elevated PSA level (>10 ng/mL after three rounds of PSA testing) and/or significantly positive findings on imaging examination (i.e., CT, MR, or bone scan) at the 3-yr follow-up period. All analyses were performed at the patient level. For patients with multiple lesions, the index lesion was defined as the one with the highest Gleason score; in cases of identical Gleason scores, the largest lesion was selected.

Supplementary Material 2

It should be noted that PI-RADS scores in this study were derived from original clinical reports, in which radiologists applied the guideline version current at the time of reporting, PI-RADS v2 for examinations prior to 2020, and PI-RADS v2.1 for those from 2020 onwards (with 5-30 years' experience in clinical work). The era-based subgroup therefore reflects actual guideline-concordant clinical practice rather than a surrogate assumption. As to the clinical PI-RADS strategy: Due to the observational nature of this study, all image review were performed on a phase-designed protocol using post-hoc analysis. Generally, after MRI acquisition, prostate images were initially assigned randomly to junior readers (with 3–5 years of radiology residency training) for preliminary evaluation using the structured PI-RADS scoring system. The initial reports were then randomly submitted to a panel of senior radiologists (each with over 10 years of experience in cross-sectional genitourinary imaging) for reassessment. In cases of disagreement in PI-RADS scores, discussion between junior and senior readers was conducted until a consensus was reached. This dual-review process aimed to minimize inter-observer variability. During image interpretation, readers had access to clinical information, including PSA levels, digital rectal examination findings, and other risk factors such as family history.

For cases where a PI-RADS score was not explicitly documented in the original records, consistent with the retrospective nature of the study, prostate images were independently reviewed by two radiologists (one junior and one senior) in accordance with PI-RADS v2.1 criteria. Both reviewers were blinded to pathological outcomes and prior imaging reports. Any discrepancies between their assessments were resolved through consensus discussion. This post-hoc reinterpretation was primarily applied to 1,478 datasets from PKUH, as the original prostate MRI scans had been acquired between July 2002 and December 2009, before the PI-RADS system had been introduced. It should be noted that MRI acquisitions from 2002-2009 may not have been fully compliant with current PI-RADS technical recommendations regarding b-value selection and dynamic contrast-enhanced parameters. This represents an inherent limitation of these historically acquired cases, which is addressed by the dual-blind standardized re-read protocol described above and further examined in a pre-specified sensitivity analysis

Supplementary Material 3

At sub-center analysis, notable heterogeneity in PI-RADS score was observed across institutions. Centers such as SCUH, ZJCH, and ZJH had a higher proportion of high PI-RADS categories (scores 4-5), with PI-RADS 5 accounting for 35.6%, 34.4%, and 34.0%, respectively, suggesting a more aggressive scoring tendency. In contrast, PKUH showed a conservative distribution, with PI-RADS 1 being the most frequent category (39.0%) and relatively fewer cases scored as PI-RADS 4-5 (15.0% and 19.6%). NJUH, TJUH, and REAC demonstrated intermediate patterns, with notable proportions of PI-RADS 3. Importantly, none of those individual centers in China presents exactly similar PI-RADS distribution with META data. These findings underscore significant inter-institutional variability in PI-RADS assessment, which may reflect differences in local imaging protocols, radiologist experience, or patient populations. Such heterogeneity emphasizes the need for standardized training and quality control in multi-center prostate MRI studies.

Supplementary Material 4

For PCa detection at cutoff of ≥3, significantly higher AUCs were observed in the older age group (≥70 years) at SCUH, NJUH, TJUH, ZJCH and ZJH (all $p < 0.001$). This pattern persisted at a cutoff of ≥4, with the same five institutions demonstrating superior performance in the ≥70 years group. For csPCa detection, age-related differences were generally less pronounced but followed a similar trend. At a cutoff of ≥3, SCUH, NJUH, TJUH, ZJCH and ZJH (all $p<0.001$) exhibited significantly higher AUCs in older patients; a similar trend was noted at a cutoff of ≥4 with minor variations. In the summary analysis, for both PCa and csPCa detection at both cutoffs, the AUC was higher in patients aged ≥70 years compared to those aged <70 years.

Supplementary Material 5

For PCa at both cutoffs (≥3 and ≥4), the >20 ng/mL group most consistently achieved the highest AUCs, with particularly significant improvements at NJUH, TJUH, PKUH, ZJH, and REAC (all $p <$ 0.001 compared to the <10 ng/mL group). For csPCa at a cutoff of ≥3, all institutions except PKUH

and REAC showed statistically significant increases in AUC with higher PSA levels. A similar pattern was observed at a cutoff of ≥4, though overall AUC values were slightly lower compared to the ≥3 threshold.

Supplementary Material 6*.*

Note: The PKUH center was excluded from this PI-RADS version subgroup analysis (v2 vs. v2.1, i.e., <2020 vs. ⩾2020) because exact examination dates at the individual case level were unavailable, precluding reliable assignment of cases to the appropriate PI-RADS version era. This exclusion was applied proactively to ensure methodological rigor of the version comparison.

For PCa detection at a cutoff of ≥3, PI-RADS v2.1 yielded significantly higher AUCs than v2 in all institutions except TJUH, with all other p-values < 0.001. At a cutoff of ≥4, significant superiority of v2.1 was maintained in SCUH, NJUH, ZJH, and ZJCH (all p < 0.001), whereas TJUH showed significantly better performance with v2 (p < 0.001). For csPCa detection at a cutoff of ≥3, v2.1 outperformed v2 across all centers (p < 0.001) except TJUH and ZJCH. At a cutoff of ≥4, v2.1 remained superior in most centers (p < 0.001), except TJUH and ZJCH (**Figure S4**). The summary results exhibited a similar trend, reinforcing the overall findings.

Table S1 Parameters of MRI scanning of DWI from the multicenter

Center	Sequences	Vendor	MRI strength	B value(sec/mm^2)	Slice thickness (mm)	Spacing between slices (mm)	Echo time(s)	Repetition time(s)
SCUH	T2WI, DWI, ADC, DCE	Siemens Skyra/Vero /GE	3T	50/70/1500/2000	2/3/4/	3/4/5	60/104	6540/7590
NJUH	T2WI, DWI, ADC, DCE	Siemens Skyra/Vero	3T	0/50/70/1500/2000	2/3/4/5	3/4/5	50/60/100	4000/4500/5000/6000
TJUH	T2WI, DWI, ADC, DCE	Siemens Skyra	3T	0/50/70/1500	3/4	3/4	55/75/100	4500/4700/4800/4900/6000
PKUH	T2WI, DWI, ADC, DCE	Philips Ingenia	1.5T	10/20/50/100/200/1000/2000	1.5/2/3/3.4/3.5/3.7/3.8/3.9/4/4.1/4.2/4.3/5	1.65/3/3.2/3.3/3.4/3.5/3.7/3.8/3.9/2.2/4/4.1/4.2/4.3/5	77/78/100	4542/4828/4898/4733/4972/ 6000
ZJH	T2WI, DWI, ADC, DCE	Philips achieva	3T	0/400/800/1500	3	3.3	62/75	2000/4342/6118/ 6173/6498/6489
SKH	T2WI, DWI, ADC, DCE	Siemens Skyra	3T	400/800/1200	3.5/4	3.5/4	72/97	3900/4342/6118/ 6173/6489/6498/7500
TZH	T2WI, DWI, ADC, DCE	Siemens Skyra and Vero	3T	0/50/800/1000/1500	3.5/4/5/5.5	3.5/4/4.8/6/6.6	62/64/74/97/104	4480/5000/5100/7500/8600
CSH	T2WI, DWI, ADC, DCE	Philips Achieva TX	3T	0, 1000, 2000	3/4/5	3/4	76/80	2750/3000
ZJCH	T2WI, DWI,	Siemens Skyra	3T	10/20/50/100/200/1000/2000	3/4	3/4	64/100	5000/7500/8000

	ADC, DCE

Abbreviation: T2WI, T2-weighted imaging; DWI, diffusion-weighted imaging; ADC, Apparent diffusion coefficient; DCE, Dynamic contrast-enhanced; SCUH, the First Affiliated Hospital of Soochow University; NJUH, the First Affiliated Hospital of Nanjing Medical University; TJUH, Tongji Hospital of Tongji Medical college; PKUH, Peking University First Hospital; ZJH, Zhangjiagang Hospital of Suzhou University; ZJCH, Zhejiang Cancer Hospital; REAC, a cross-district re-alliancing center.

Table S2. Sensitivity Analysis: Diagnostic Performance with and without PKUH, with Statistical Comparison

	Cutoff	Full cohort (n = 20,429)			PKUH-excluded (n = 18,951)			PKUH only (n = 1,478)			PKUH vs Non-PKUH		
**Outcome**		**AUC (95% CI)**	**SEN % (95% CI)**	**SPE % (95% CI)**	**AUC (95% CI)**	**SEN % (95% CI)**	**SPE % (95% CI)**	**AUC (95% CI)**	**SEN % (95% CI)**	**SPE % (95% CI)**	**Δ AUC / *p***	**Δ SEN / *p***	**Δ SPE / *p***
**PCa**	≥3	0.838 (0.833–0.843)	90.9 (90.3–91.4)	51.6 (50.6–52.6)	0.831 (0.825–0.837)	91.0 (90.4–91.5)	48.9 (47.9–50.0)	0.878 (0.858–0.898)	89.6 (86.3–92.1)	73.7 (71.0–76.3)	**+0.047** *p* = <0.001 *	**-1.4%** *p* = 0.331	**+24.8%** *p* = <0.001 *
	≥4	0.838 (0.833–0.843)	79.0 (78.3–79.8)	78.6 (77.8–79.4)	0.831 (0.825–0.837)	79.0 (78.2–79.8)	78.0 (77.1–78.9)	0.878 (0.858–0.898)	79.9 (75.8–83.4)	84.0 (81.7–86.1)	**+0.047** *p* = <0.001 *	**+0.8%** *p* = 0.672	**+6.0%** *p* = <0.001 *
**csPCa**	≥3	0.845 (0.840–0.850)	92.8 (92.3–93.3)	49.1 (48.1–50.0)	0.840 (0.835–0.846)	92.8 (92.3–93.3)	46.7 (45.7–47.6)	0.875 (0.854–0.896)	92.5 (89.3–94.9)	69.9 (67.2–72.5)	**+0.035** *p* = 0.002 *	**-0.3%** *p* = 0.844	**+23.3%** *p* = <0.001 *
	≥4	0.845 (0.840–0.850)	82.5 (81.7–83.3)	76.0 (75.2–76.8)	0.840 (0.835–0.846)	82.4 (81.6–83.2)	75.5 (74.6–76.3)	0.875 (0.854–0.896)	84.5 (80.3–87.9)	80.7 (78.3–82.9)	**+0.035** *p* = 0.002 *	**+2.0%** *p* = 0.324	**+5.2%** *p* = <0.001 *

Notes: AUC, area under the ROC curve; CI, 95% confidence interval; csPCa, clinically significant prostate cancer; PCa, prostate cancer; SEN, sensitivity; SPE, specificity.
PKUH = Peking University First Hospital (n = 1,478; 7.2% of total cohort); cases acquired July 2002–December 2009 (pre-PI-RADS era), retrospectively scored by two blinded radiologists using PI-RADS v2.1 with consensus resolution
AUC and 95% CI: DeLong's method. Sensitivity and specificity 95% CI: Wilson score interval. Statistical comparison (PKUH vs non-PKUH): two-proportion z-test for SEN and SPE; independent DeLong test for AUC.
* $p < 0.05$ (red shading = statistically significant; green shading = not significant). PKUH showed significantly higher AUC and SPE vs non-PKUH centers, attributable to substantially lower disease prevalence at PKUH (PCa: 29.2%; csPCa 23.5%) vs non-PKUH (PCa: 53.7%; csPCa: 48.5%), a well-known prevalence effect on specificity. Sensitivity did not differ significantly across all scenarios (all $p > 0.30$). Despite these statistically significant differences for PKUH vs non PKUH, the absolute impact of including PKUH on the full cohort pooled estimates is negligible (ΔAUC ≤ 0.007; ΔSEN ≤ 0.1%; ΔSPE ≤ 2.7%), because PKUH constitutes only 7.2% of the total cohort. The primary conclusions of this stud are therefore robust to the inclusion of PKUH.

Table S3.

The baseline characteristics of multicenter patients.

Variable	SCUH, n = 5880			NJUH, n = 5142			TJUH, n = 4562			PKUH, n = 1478			ZJH, n=1299			REAC, n = 1173			ZJCH, n=895		
	PCa	Benign	*P*-value	PCa	Benign	*P*-value	PCa	Benign	*P*-value	PCa	Benign	*P*-value	PCa	Benign	*P*-value	PCa	Benign	*P*-value	PCa	Benign	*P*-value
No. of subjects	3258	2622		2923	2219		2229	2333		432	1046		696	603		486	687		582	313	
Age (y), median (IRQ)	72.0 (67.0, 76.0)	67.0 (61.2, 72.0)	⩽ 0.0001	71.0 (65.0, 76.0)	66.0 (60.0, 71.0)	⩽ 0.0001	73.0 (68.0, 79.0)	70.0 (64.0, 75.0)	⩽ 0.0001	72.0 (67.0, 76.0)	70.0 (65.0, 75.0)		73.0 (69.0, 78.0)	70.0 (65.0, 75.0)	⩽ 0.0001	73.0 (67.0, 77.0)	69.0 (64.0, 74.0)	⩽ 0.0001	70.0 (65.0, 75.0)	67.0 (61.0, 72.0)	⩽ 0.0001
< 70 y, n (%)	1279 (39.3%)	1649 (62.9%)	⩽ 0.0001	1298 (44.4%)	1515 (68.3%)	⩽0.0001	723 (32.4%)	1099 (47.1%)	⩽0.0001	162 (37.5%)	495 (47.3%)	⩽0.0001	205 (29.5%)	267 (44.3%)	⩽ 0.0001	175 (36.0%)	376 (54.7%)	⩽ 0.0001	275 (47.3%)	198(63.3%)	⩽ 0.0001
⩾ 70 y, n (%)	1979 (60.7%)	973 (37.1%)	⩽ 0.0001	1625 (55.6%)	704 (31.7%)	⩽0.0001	1506 (67.6%)	1234 (52.9%)	⩽0.0001	270 (62.5%)	551 (52.7%)	⩽0.0001	491 (70.5%)	336 (55.7%)	⩽ 0.0001	311 (64.0%)	311 (45.3%)	⩽ 0.0001	307 (52.7%)	115(36.7%)	⩽ 0.0001
Prostate Zone, n	3258	2622					2229	2333					696	603							
PZ, n (%)	1516 (46.5%)	1012 (38.6%)	⩽ 0.0001				600 (26.9%)	320 (13.7%)	⩽ 0.0001				256 (36.8%)	232 (38.5%)	0.568						
TZ/ AFMS/CZ, n (%)	1742 (53.5%)	1610 (61.4%)	⩽ 0.0001				1629 (73.1%)	2013 (86.3%)	⩽ 0.0001				440 (63.2%)	371 (61.5%)	0.568						
ISUP grade, n (%)	3258	2622		2923	2219		2229	2333		432	101		696	603		486	687		582	313	
GG0 (Benign)		2622 (100%)			2219 (100.0%)			2333 (100.0%)			1046 (100.0%)			603 (100.0%)			687 (100.0%)			313 (100.0%)	

GG1	247 (7.6%)			305 (10.4%)			200 (8.9%)			82 (19.0%)			86 (12.4%)			92 (18.9%)			67 (11.5%)		
GG2	822 (25.2%)			735 (25.1%)			442 (19.8%)			80 (18.5%)			119(17.1%)			105 (21.6%)			118 (20.3%)		
GG3	928 (28.5%)			867 (29.7%)			490 (22.0%)			36 (8.3%)			136(19.5%)			79 (16.3%)			138 (23.7%)		
GG4	591 (18.1%)			636 (21.8%)			485 (21.8%)			60 (13.9%)			186(26.7%)			109 (22.4%)			127 (21.8%)		
GG5	670 (20.6%)			380 (13.0%)			612 (27.5%)			174 (40.3%)			169(24.3%)			101 (20.8%)			132 (22.7%)		

Notes: Unless indicated otherwise, data are numbers of patients with percentage in parentheses. *P*-value was evaluated by two-tailed t-test with unequal variance. Gleason grade (GG) is according to the 2014 International Society of Urological Pathology (ISUP) standards. PCa = prostate cancer; PZ = peripheral zone; TZ = transition zone; CZ = center zone; AFMS = anterior fibromuscular stroma; SCUH, the First Affiliated Hospital of Soochow University; NJUH, the First Affiliated Hospital of Nanjing Medical University; TJUH, Tongji Hospital of Tongji Medical college; PKUH, Peking University First Hospital; ZJH, Zhangjiagang Hospital of Suzhou University; ZJCH, Zhejiang Cancer Hospital; REAC, a cross-district re-alliancing center.

Table S4 The diagnosis performance of PI-RADS in predicting PCa and csPCa in multicenter

Centers	Outcome	Cutoff	No. of patients	SPE	PPV	NPV
**SCUH**	**PCa**	**PI-RADS ⩾3**	5880	37.22 (35.4-39.1)	64.7 (63.3-66.0)	79.9 (77.6-82.1)
		**PI-RADS ⩾4**		66.74 (64.9-68.5)	74.9 (73.4-76.3)	72.7 (70.9-74.5)
	**csPCa**	**PI-RADS ⩾3**		35.83 (34.1-37.6)	60.5 (59.1-61.9)	84.2 (82.0-86.2)
		**PI-RADS ⩾4**		65.32 (63.5-67.1)	71.4 (69.8-72.9)	77.9 (76.2-79.5)
**NJUH**	**PCa**	**PI-RADS ⩾3**	5142	47.68 (45.6-49.8)	70.08 (68.6-71.5)	83.93 (81.9-86.0)
		**PI-RADS ⩾4**		78.72 (77.0-80.4)	83.83 (82.5-85.2)	78.62 (76.9-80.3)
	**csPCa**	**PI-RADS ⩾3**		44.84 (42.9-46.8)	64.11 (62.6-65.6)	89.79 (88.1-91.5)
		**PI-RADS ⩾4**		75.41 (73.7-77.1)	78.73 (77.2-80.2)	85.67 (84.2-87.1)
**TJUH**	**PCa**	**PI-RADS ⩾3**	4562	60.01 (58.0-62.0)	67.7 (65.9-69.4)	83.5 (81.6-85.2)
		**PI-RADS ⩾4**		85.30 (83.8-86.7)	83.1 (81.4-84.7)	78.5 (76.8-80.1)
	**csPCa**	**PI-RADS ⩾3**		58.29 (56.3-60.2)	63.6 (61.8-65.3)	87.6 (85.9-89.1)
		**PI-RADS ⩾4**		83.25 (81.7-84.7)	79.2 (77.3-80.9)	82.8 (81.2-84.2)
**ZJH**	**PCa**	**PI-RADS ⩾3**	1299	46.77 (42.7-50.8)	66.1 (63.0-69.1)	80.1 (75.6-84.2)
		**PI-RADS ⩾4**		81.26 (77.9-84.3)	82.1 (78.8-85.0)	73.2 (69.7-76.6)
	**csPCa**	**PI-RADS ⩾3**		44.41 (40.7-48.2)	59.6 (56.4-62.7)	86.9 (83.0-90.3)
		**PI-RADS ⩾4**		78.96 (75.7-81.9)	77.0 (73.5-80.2)	81.3 (78.2-84.2)
**PKUH**	**PCa**	**PI-RADS ⩾3**	1478	73.62 (71.0-76.3)	58.41 (54.7-62.2)	94.28 (92.7-95.9)
		**PI-RADS ⩾4**		83.91 (81.7-86.1)	67.25 (63.2-71.3)	90.83 (89.0-92.6)
	**csPCa**	**PI-RADS ⩾3**		69.84 (67.2-72.5)	48.65 (44.9-52.4)	96.59 (95.4-97.8)
		**PI-RADS ⩾4**		80.60 (78.3-82.9)	57.37 (53.1-61.6)	94.23 (92.8-95.7)
**REAC**	**PCa**	**PI-RADS ⩾3**	1173	58.1 (54.4-61.7)	61.0 (57.4-64.4)	91.7 (88.8-94.0)
		**PI-RADS ⩾4**		90.4 (88.0-92.4)	84.2 (80.5-87.4)	82.4 (79.5-84.9)
	**csPCa**	**PI-RADS ⩾3**	1173	53.9 (50.4-57.4)	51.4 (47.8-54.9)	96.6 (94.4-97.9)
		**PI-RADS ⩾4**		86.5 (83.9-88.7)	74.9 (70.6-78.9)	89.4 (87.0-91.4)
**ZJCH**	**PCa**	**PI-RADS ⩾3**	895	57.51 (51.8-63.1)	78.7 (75.3-81.8)	66.4 (60.5-72.0)
		**PI-RADS ⩾4**		78.91 (74.0-83.3)	86.8 (83.5-89.6)	62.5 (57.6-67.3)
	**csPCa**	**PI-RADS ⩾3**	895	52.37 (47.2-57.5)	71.0 (67.3-74.5)	73.4 (67.8-78.6)
		**PI-RADS ⩾4**		71.58 (66.8-76.1)	78.4 (74.5-81.9)	68.9 (64.0-73.4)

Notes: PCa, prostate cancer; csPCa, clinically significant prostate cancer; SCUH, the First Affiliated Hospital of Soochow University; NJUH, the First Affiliated Hospital of Nanjing Medical University; TJUH, Tongji Hospital of Tongji Medical college; PKUH, Peking University First Hospital; ZJH, Zhangjiagang Hospital of Suzhou University; ZJCH, Zhejiang Cancer Hospital; REAC, a cross-district re-alliancing center.

Table S5 Subgroup analysis of PI-RADS in predicting PCa and csPCa by age (<70y and ≥70 y)

Centers	Outcome	Age	Cutoff	No.	Positive	Negative	AUC	SEN	SPE	PPV	NPV
**SCUH**	**PCa**	**＜70y**	**PI-RADS ≥3**	2928	1279 (43.7%)	1649 (56.3%)	0.767 (0.751-0.784)	0.910 (0.893-0.925)	0.372 (0.349-0.396)	0.529 (0.508-0.550)	0.842 (0.814-0.868)
			**PI-RADS ≥4**				0.767 (0.751-0.784)	0.758 (0.733-0.781)	0.687 (0.664-0.709)	0.653 (0.628-0.677)	0.785 (0.763-0.806)
		**≥70y**	**PI-RADS ≥3**	2952	1979 (67.0%)	973 (33.0%)	0.795 (0.778-0.811)	0.934 (0.922-0.945)	0.372 (0.342-0.403)	0.752 (0.734-0.769)	0.736 (0.694-0.774)
			**PI-RADS ≥4**				0.795 (0.778-0.811)	0.825 (0.808-0.842)	0.634 (0.603-0.664)	0.821 (0.803-0.838)	0.641 (0.609-0.671)
	**csPCa**	**＜70y**	**PI-RADS ≥3**	2928	1163 (39.7%)	1765 (60.3%)	0.780 (0.763-0.797)	0.925 (0.909-0.940)	0.364 (0.341-0.387)	0.489 (0.468-0.510)	0.881 (0.855-0.903)
			**PI-RADS ≥4**				0.780 (0.763-0.797)	0.788 (0.764-0.812)	0.678 (0.656-0.700)	0.618 (0.592-0.642)	0.830 (0.809-0.849)
		**≥70y**	**PI-RADS ≥3**	2952	1848 (62.6%)	1104 (37.4%)	0.799 (0.783-0.815)	0.943 (0.931-0.953)	0.350 (0.321-0.379)	0.708 (0.690-0.726)	0.785 (0.746-0.820)
			**PI-RADS ≥4**				0.799 (0.783-0.815)	0.845 (0.828-0.861)	0.613 (0.584-0.642)	0.785 (0.767-0.803)	0.703 (0.673-0.732)
**NJUH**	**PCa**	**＜70y**	**PI-RADS ≥3**	2813	1299 (46.2%)	1514 (53.8%)	0.847 (0.833-0.861)	0.916 (0.900-0.930)	0.491 (0.466-0.517)	0.607 (0.585-0.629)	0.872 (0.848-0.893)
			**PI-RADS ≥4**				0.847 (0.832-0.860)	0.808 (0.785-0.828)	0.796 (0.775-0.815)	0.772 (0.749-0.794)	0.828 (0.808-0.847)
		**≥70y**	**PI-RADS ≥3**	2329	1624 (69.7%)	705 (30.3%)	0.869 (0.854-0.882)	0.943 (0.931-0.954)	0.445 (0.409-0.482)	0.797 (0.778-0.814)	0.773 (0.730-0.811)
			**PI-RADS ≥4**				0.869 (0.853-0.884)	0.862 (0.844-0.878)	0.770 (0.738-0.800)	0.896 (0.880-0.910)	0.708 (0.675-0.739)
	**csPCa**	**＜70y**	**PI-RADS ≥3**	2813	1132 (40.2%)	1681 (59.8%)	0.866 (0.853-0.879)	0.942 (0.926-0.954)	0.468 (0.444-0.492)	0.544 (0.522-0.566)	0.923 (0.903-0.939)
			**PI-RADS ≥4**				0.866 (0.853-0.879)	0.859 (0.837-0.878)	0.770 (0.750-0.790)	0.716 (0.691-0.739)	0.890 (0.873-0.905)
		**≥70y**	**PI-RADS ≥3**	2329	1485 (63.8%)	844 (36.2%)	0.867 (0.852-0.881)	0.959 (0.948-0.968)	0.409 (0.376-0.442)	0.741 (0.720-0.760)	0.850 (0.812-0.881)
			**PI-RADS ≥4**				0.867 (0.853-0.882)	0.894 (0.878-0.909)	0.723 (0.692-0.752)	0.850 (0.832-0.867)	0.795 (0.765-0.822)

**TJUH**	**PCa**	**＜70y**	**PI-RADS ≥3**	1822	723 (39.7%)	1099 (60.3%)	0.831 (0.812-0.850)	0.869 (0.842-0.892)	0.579 (0.549-0.608)	0.576 (0.546-0.605)	0.870 (0.843-0.894)
			**PI-RADS ≥4**				0.831 (0.812-0.850)	0.737 (0.704-0.769)	0.846 (0.824-0.867)	0.759 (0.726-0.790)	0.830 (0.807-0.852)
		**≥70y**	**PI-RADS ≥3**	2740	1506 (55.0%)	1234 (45.0%)	0.848(0.834-0.862)	0.879 (0.862-0.895)	0.619 (0.591-0.646)	0.738 (0.717-0.758)	0.808 (0.781-0.832)
			**PI-RADS ≥4**				0.848 (0.834-0.862)	0.764 (0.742-0.786)	0.859 (0.838-0.878)	0.869 (0.849-0.886)	0.749 (0.726-0.772)
	**csPCa**	**＜70y**	**PI-RADS ≥3**	1822	660 (36.2%)	1162 (63.8%)	0.846 (0.827-0.865)	0.894 (0.868-0.916)	0.569 (0.540-0.598)	0.541 (0.511-0.571)	0.904 (0.881-0.925)
			**PI-RADS ≥4**				0.846 (0.827-0.865)	0.771 (0.737-0.803)	0.834 (0.811-0.855)	0.725 (0.690-0.758)	0.865 (0.844-0.885)
		**≥70y**	**PI-RADS ≥3**	2740	1382 (50.4%)	1358 (49.6%)	0.855 (0.840-0.868)	0.900 (0.883-0.915)	0.595 (0.568-0.621)	0.693 (0.672-0.715)	0.854 (0.830-0.876)
			**PI-RADS ≥4**				0.855 (0.840-0.868)	0.793 (0.771-0.814)	0.831 (0.810-0.851)	0.827 (0.806-0.847)	0.798 (0.776-0.819)
**PKUH**	**PCa**	**＜70y**	**PI-RADS ≥3**	657	162 (24.66%)	495 (75.34%)	0.886 (0.850-0.918)	0.889 (0.831-0.929)	0.788 (0.750-0.822)	0.578 (0.516-0.638)	0.956 (0.931-0.972)
			**PI-RADS ≥4**				0.886 (0.850-0.918)	0.759 (0.688-0.819)	0.887 (0.856-0.912)	0.687 (0.616-0.751)	0.918 (0.890-0.940)
		**≥70y**	**PI-RADS ≥3**	821	270 (32.89%)	551 (67.11%)	0.868 (0.843-0.893)	0.900 (0.858-0.930)	0.691 (0.652-0.729)	0.588 (0.540-0.635)	0.934 (0.905-0.954)
			**PI-RADS ≥4**				0.868 (0.843-0.893)	0.822 (0.772-0.863)	0.799 (0.763-0.830)	0.667 (0.614-0.715)	0.902 (0.872-0.925)
	**csPCa**	**＜70y**	**PI-RADS ≥3**	657	134 (20.4%)	523 (79.6%)	0.894 (0.860-0.928)	0.910 (0.850-0.948)	0.757 (0.719-0.792)	0.490 (0.428-0.552)	0.971 (0.949-0.983)
			**PI-RADS ≥4**				0.894 (0.860-0.928)	0.813 (0.739-0.870)	0.866 (0.834-0.893)	0.609 (0.536-0.677)	0.948 (0.924-0.964)
		**≥70y**	**PI-RADS ≥3**	821	214 (26.1%)	607 (73.9%)	0.854 (0.826-0.879)	0.935 (0.893-0.961)	0.649 (0.610-0.686)	0.484 (0.436-0.532)	0.966 (0.943-0.979)
			**PI-RADS ≥4**				0.854 (0.826-0.879)	0.864 (0.812-0.904)	0.756 (0.720-0.789)	0.556 (0.502-0.608)	0.941 (0.916-0.958)
**ZJH**	**PCa**	**＜70y**	**PI-RADS ≥3**	472	205 (43.4%)	267 (56.6%)	0.771 (0.729-0.813)	0.854 (0.798-0.899)	0.472 (0.411-0.534)	0.554 (0.497-0.609)	0.808 (0.737-0.866)

			PI-RADS ≥4				0.771 (0.729-0.813)	0.600 (0.529-0.668)	0.831 (0.781-0.874)	0.732 (0.658-0.797)	0.730 (0.677-0.779)
		≥70y	PI-RADS ≥3	827	491 (59.4%)	336 (40.6%)	0.849 (0.823-0.875)	0.919 (0.891-0.941)	0.464 (0.410-0.519)	0.715 (0.678-0.750)	0.796 (0.733-0.850)
			PI-RADS ≥4				0.849 (0.823-0.875)	0.802 (0.764-0.837)	0.798 (0.751-0.839)	0.853 (0.817-0.884)	0.734 (0.686-0.779)
	**csPCa**	**＜70y**	**PI-RADS ≥3**	472	167 (35.4%)	305 (64.6%)	0.816 (0.776-0.857)	0.904 (0.849-0.944)	0.459 (0.402-0.517)	0.478 (0.422-0.534)	0.897 (0.839-0.940)
			**PI-RADS ≥4**				0.816 (0.776-0.857)	0.695 (0.619-0.763)	0.830 (0.783-0.870)	0.690 (0.615-0.759)	0.832 (0.785-0.872)
		**≥70y**	**PI-RADS ≥3**	827	443 (53.6%)	384 (46.4%)	0.849 (0.823-0.875)	0.932 (0.905-0.954)	0.432 (0.382-0.484)	0.655 (0.616-0.692)	0.847 (0.789-0.894)
			**PI-RADS ≥4**				0.849 (0.823-0.875)	0.833 (0.795-0.866)	0.758 (0.712-0.800)	0.799 (0.759-0.834)	0.797 (0.752-0.837)
**REAC**	**PCa**	**＜70y**	**PI-RADS ≥3**	551	175 (31.8%)	376 (68.2%)	0.878 (0.766–0.989)	0.914 (0.863–0.947)	0.641 (0.591–0.688)	0.542 (0.485–0.598)	0.941 (0.906–0.964)
			**PI-RADS ≥4**				0.878 (0.766–0.989)	0.686 (0.614–0.750)	0.918 (0.885–0.941)	0.795 (0.723–0.851)	0.863 (0.825–0.893)
		**≥70y**	**PI-RADS ≥3**	622	311 (50.0%)	311 (50.0%)	0.868 (0.786–0.950)	0.932 (0.899–0.955)	0.508 (0.453–0.563)	0.655 (0.609–0.697)	0.883 (0.827–0.922)
			**PI-RADS ≥4**				0.868 (0.786–0.950)	0.749 (0.698–0.794)	0.887 (0.848–0.918)	0.869 (0.824–0.905)	0.780 (0.734–0.820)
	**csPCa**	**＜70y**	**PI-RADS ≥3**	551	139 (25.2%)	412 (74.8%)	0.913 (0.776–1.000)	0.971 (0.928–0.989)	0.612 (0.564–0.657)	0.458 (0.402–0.515)	0.984 (0.961–0.994)
			**PI-RADS ≥4**				0.913 (0.776–1.000)	0.777 (0.701–0.838)	0.896 (0.862–0.922)	0.715 (0.639–0.781)	0.922 (0.892–0.945)
		**≥70y**	**PI-RADS ≥3**	622	255 (41.0%)	367 (59.0%)	0.877 (0.784–0.969)	0.957 (0.924–0.976)	0.458 (0.407–0.509)	0.551 (0.504–0.596)	0.939 (0.893–0.965)
			**PI-RADS ≥4**				0.877 (0.784–0.969)	0.808 (0.755–0.851)	0.831 (0.789–0.866)	0.769 (0.715–0.815)	0.862 (0.822–0.894)
**ZJH**	**PCa**	**＜70y**	**PI-RADS ≥3**	472	205 (43.4%)	267 (56.6%)	0.775 (0.734-0.817)	0.807 (0.756-0.852)	0.596 (0.524-0.665)	0.735 (0.682-0.784)	0.690 (0.615-0.758)
			**PI-RADS ≥4**				0.775 (0.734-0.817)	0.691 (0.633-0.745)	0.823 (0.763-0.874)	0.844 (0.790-0.889)	0.657 (0.595-0.716)
		**≥70y**	**PI-RADS**	827	491	336	0.803 (0.757-	0.876 (0.834-	0.539 (0.444-	0.835 (0.790-	0.620 (0.517-

			**≥3**		(59.4%)	(40.6%)		0.848)		0.911)		0.632)		0.874)		0.715)
			**PI-RADS ≥4**				0.803	(0.757-0.848)	0.795	(0.745-0.839)	0.730	(0.640-0.809)	0.887	(0.844-0.922)	0.571	(0.487-0.653)
	**csPCa**	**＜70y**	**PI-RADS ≥3**	472	167 (35.4%)	305 (64.6%)	0.770	(0.729-0.812)	0.828	(0.774-0.873)	0.553	(0.487-0.618)	0.652	(0.596-0.706)	0.760	(0.689-0.822)
			**PI-RADS ≥4**				0.770	(0.729-0.812)	0.714	(0.652-0.771)	0.766	(0.707-0.819)	0.756	(0.694-0.810)	0.726	(0.666-0.780)
		**≥70y**	**PI-RADS ≥3**	827	443 (53.6%)	384 (46.4%)	0.771	(0.726-0.817)	0.888	(0.845-0.923)	0.476	(0.392-0.560)	0.764	(0.714-0.809)	0.690	(0.590-0.779)
			**PI-RADS ≥4**				0.771	(0.726-0.817)	0.801	(0.750-0.847)	0.634	(0.551-0.713)	0.807	(0.756-0.852)	0.626	(0.542-0.704)
**SUM**	**PCa**	**＜70y**	**PI-RADS ≥3**	9716	4118 (42.4%)	5598 (57.6%)	0.821	(0.812-0.829)	0.894	(0.885-0.903)	0.513	(0.499-0.526)	0.574	(0.562-0.586)	0.868	(0.856-0.879)
			**PI-RADS ≥4**				0.821	(0.813-0.829)	0.754	(0.741-0.767)	0.793	(0.782-0.803)	0.728	(0.714-0.741)	0.814	(0.804-0.825)
		**≥70y**	**PI-RADS ≥3**	10713	6488 (60.6%)	4225 (39.4%)	0.847	(0.840-0.854)	0.918	(0.911-0.925)	0.520	(0.505-0.535)	0.746	(0.736-0.755)	0.806	(0.790-0.820)
			**PI-RADS ≥4**				0.847	(0.840-0.854)	0.813	(0.804-0.823)	0.778	(0.765-0.790)	0.849	(0.840-0.858)	0.731	(0.718-0.744)
	**csPCa**	**＜70y**	**PI-RADS ≥3**	9716	3633 (37.4%)	6083 (62.6%)	0.837	(0.828-0.845)	0.919	(0.909-0.927)	0.494	(0.482-0.507)	0.520	(0.508-0.533)	0.910	(0.900-0.920)
			**PI-RADS ≥4**				0.837	(0.829-0.845)	0.799	(0.785-0.811)	0.775	(0.765-0.786)	0.680	(0.666-0.694)	0.866	(0.856-0.874)
		**≥70y**	**PI-RADS ≥3**	10713	5904 (55.1%)	4809 (44.9%)	0.846	(0.838-0.853)	0.934	(0.927-0.940)	0.486	(0.472-0.500)	0.690	(0.680-0.700)	0.857	(0.843-0.869)
			**PI-RADS ≥4**				0.846	(0.839-0.854)	0.841	(0.832-0.851)	0.741	(0.728-0.753)	0.799	(0.789-0.809)	0.792	(0.780-0.804)

Notes: SCUH, the First Affiliated Hospital of Soochow University; NJUH, the First Affiliated Hospital of Nanjing Medical University; TJUH, Tongji Hospital of Tongji Medical college; PKUH, Peking University First Hospital; ZJH, Zhangjiagang Hospital of Suzhou University; ZJCH, Zhejiang Cancer Hospital; REAC, a cross-district re-alliancing center.

Table S6 Subgroup analysis of PI-RADS in predicting PCa and csPCa by PSA (PSA<10, 10≤PSA≤20 and PSA>20)

Centers	Outcome	PSA	Cutoff	No.	Positive	Negative	AUC	SEN	SPE	PPV	NPV
**NJUH**	**PCa**	**PSA<10**	**PI-RADS ≥3**	2403	884 (36.8%)	1519 (63.2%)	0.804 (0.787-0.822)	0.894 (0.872-0.912)	0.481 (0.456-0.506)	0.501 (0.476-0.525)	0.886 (0.863-0.906)
			**PI-RADS ≥4**				0.804 (0.787-0.822)	0.752 (0.723-0.780)	0.793 (0.772-0.812)	0.679 (0.649-0.707)	0.846 (0.826-0.864)
		**10≤PSA≤20**	**PI-RADS ≥3**	1375	857 (62.3%)	518 (37.7%)	0.837 (0.816-0.857)	0.914 (0.893-0.931)	0.483 (0.440-0.526)	0.745 (0.718-0.770)	0.772 (0.723-0.814)
			**PI-RADS ≥4**				0.837 (0.816-0.857)	0.806 (0.778-0.831)	0.805 (0.769-0.837)	0.872 (0.847-0.894)	0.715 (0.677-0.750)
		**PSA>20**	**PI-RADS ≥3**	1364	1182 (86.7%)	182 (13.3%)	0.905 (0.881-0.926)	0.972 (0.961-0.980)	0.423 (0.354-0.496)	0.916 (0.900-0.930)	0.700 (0.609-0.778)
			**PI-RADS ≥4**				0.905 (0.881-0.926)	0.925 (0.908-0.938)	0.698 (0.628-0.760)	0.952 (0.938-0.963)	0.588 (0.521-0.652)
	**csPCa**	**PSA<10**	**PI-RADS ≥3**	2403	690 (28.7%)	1713 (71.3%)	0.824 (0.807-0.842)	0.925 (0.903-0.942)	0.451 (0.428-0.475)	0.404 (0.380-0.429)	0.937 (0.918-0.952)
			**PI-RADS ≥4**				0.824 (0.807-0.842)	0.822 (0.791-0.848)	0.759 (0.738-0.779)	0.579 (0.547-0.609)	0.914 (0.898-0.927)
		**10≤PSA≤20**	**PI-RADS ≥3**	1375	777 (56.5%)	598 (43.5%)	0.838 (0.816-0.858)	0.934 (0.915-0.950)	0.457 (0.417-0.497)	0.691 (0.662-0.718)	0.843 (0.799-0.878)
			**PI-RADS ≥4**				0.838 (0.816-0.858)	0.844 (0.817-0.868)	0.773 (0.737-0.804)	0.828 (0.800-0.853)	0.792 (0.758-0.823)
		**PSA>20**	**PI-RADS ≥3**	1364	1150 (84.3%)	214 (15.7%)	0.897 (0.873-0.919)	0.979 (0.969-0.986)	0.402 (0.338-0.469)	0.898 (0.880-0.913)	0.782 (0.696-0.849)
			**PI-RADS ≥4**				0.897 (0.873-0.919)	0.937 (0.921-0.949)	0.668 (0.603-0.728)	0.938 (0.923-0.951)	0.662 (0.597-0.722)
**TJUH**	**PCa**	**PSA<10**	**PI-RADS ≥3**	1830	542 (29.6%)	1288 (70.4%)	0.748 (0.725-0.771)	0.758 (0.721-0.792)	0.621 (0.594-0.647)	0.457 (0.425-0.490)	0.859 (0.835-0.880)
			**PI-RADS ≥4**				0.748 (0.725-0.771)	0.539 (0.497-0.580)	0.876 (0.857-0.893)	0.646 (0.601-0.689)	0.819 (0.797-0.838)
		**10≤PSA≤20**	**PI-RADS ≥3**	1162	487 (41.9%)	675 (58.1%)	0.803 (0.780-0.829)	0.821 (0.785-0.853)	0.606 (0.569-0.642)	0.601 (0.563-0.637)	0.825 (0.789-0.856)
			**PI-RADS ≥4**				0.803 (0.780-0.829)	0.688 (0.645-0.727)	0.856 (0.828-0.881)	0.775 (0.734-0.812)	0.792 (0.761-0.820)
		**PSA>20**	**PI-RADS ≥3**	1570	1200 (76.4%)	370 (23.6%)	0.860 (0.837-0.882)	0.951 (0.937-0.962)	0.516 (0.465-0.567)	0.864 (0.845-0.882)	0.764 (0.708-0.812)
			**PI-RADS ≥4**				0.860 (0.837-0.882)	0.881 (0.861-0.898)	0.768 (0.722-0.808)	0.925 (0.908-0.939)	0.665 (0.619-0.708)

	**csPCa**	**PSA<10**	**PI-RADS ≥3**	1830	437 (23.9%)	1393 (76.1%)	0.764 (0.739-0.791)	0.796 (0.756-0.831)	0.604 (0.579-0.630)	0.387 (0.356-0.419)	0.904 (0.884-0.922)
			**PI-RADS ≥4**				0.764 (0.739-0.791)	0.577 (0.530-0.622)	0.856 (0.837-0.874)	0.558 (0.511-0.603)	0.866 (0.847-0.883)
		**10≤PSA≤20**	**PI-RADS ≥3**	1162	430 (37.0%)	732 (63.0%)	0.808 (0.782-0.834)	0.842 (0.804-0.873)	0.585 (0.549-0.620)	0.544 (0.506-0.581)	0.863 (0.830-0.890)
			**PI-RADS ≥4**				0.808 (0.782-0.834)	0.719 (0.674-0.759)	0.832 (0.803-0.857)	0.715 (0.671-0.756)	0.834 (0.806-0.859)
		**PSA>20**	**PI-RADS ≥3**	1570	1175 (74.8%)	395 (25.2%)	0.854 (0.831-0.876)	0.957 (0.943-0.967)	0.504 (0.455-0.553)	0.852 (0.831-0.870)	0.796 (0.742-0.841)
			**PI-RADS ≥4**				0.854 (0.831-0.876)	0.889 (0.869-0.905)	0.749 (0.704-0.790)	0.913 (0.896-0.928)	0.693 (0.648-0.735)
**ZJH**	**PCa**	**PSA<10**	**PI-RADS ≥3**	415	170 (41.0%)	245 (59.0%)	0.776 (0.730-0.817)	0.853 (0.792-0.898)	0.473 (0.412-0.536)	0.529 (0.470-0.587)	0.823 (0.751-0.877)
			**PI-RADS ≥4**				0.776 (0.730-0.817)	0.641 (0.567-0.709)	0.800 (0.745-0.845)	0.690 (0.614-0.757)	0.763 (0.707-0.811)
		**10≤PSA≤20**	**PI-RADS ≥3**	438	206 (47.0%)	232 (53.0%)	0.797 (0.754-0.836)	0.888 (0.838-0.924)	0.474 (0.411-0.538)	0.600 (0.544-0.653)	0.827 (0.754-0.882)
			**PI-RADS ≥4**				0.797 (0.754-0.836)	0.670 (0.603-0.730)	0.806 (0.750-0.852)	0.754 (0.687-0.811)	0.733 (0.676-0.784)
		**PSA>20**	**PI-RADS ≥3**	446	320 (71.7%)	126 (28.3%)	0.863 (0.823-0.897)	0.931 (0.898-0.954)	0.444 (0.361-0.532)	0.810 (0.767-0.847)	0.718 (0.610-0.806)
			**PI-RADS ≥4**				0.863 (0.823-0.897)	0.844 (0.800-0.879)	0.849 (0.776-0.901)	0.934 (0.900-0.958)	0.682 (0.605-0.749)
	**csPCa**	**PSA<10**	**PI-RADS ≥3**	415	132 (31.8%)	283 (68.2%)	0.806 (0.759-0.853)	0.886 (0.821-0.930)	0.445 (0.388-0.503)	0.427 (0.370-0.486)	0.894 (0.832-0.934)
			**PI-RADS ≥4**				0.806 (0.759-0.853)	0.735 (0.654-0.803)	0.784 (0.733-0.828)	0.614 (0.536-0.686)	0.864 (0.816-0.900)
		**10≤PSA≤20**	**PI-RADS ≥3**	438	176 (40.2%)	262 (59.8%)	0.809 (0.767-0.848)	0.915 (0.864-0.948)	0.450 (0.391-0.511)	0.528 (0.472-0.583)	0.887 (0.822-0.930)
			**PI-RADS ≥4**				0.809 (0.767-0.848)	0.710 (0.639-0.772)	0.779 (0.725-0.825)	0.683 (0.612-0.746)	0.800 (0.747-0.844)
		**PSA>20**	**PI-RADS ≥3**	446	302 (67.7%)	144 (32.3%)	0.865 (0.826-0.899)	0.947 (0.916-0.967)	0.431 (0.353-0.512)	0.777 (0.732-0.817)	0.795 (0.692-0.870)
			**PI-RADS ≥4**				0.865 (0.826-0.899)	0.871 (0.828-0.904)	0.819 (0.749-0.874)	0.910 (0.871-0.938)	0.752 (0.679-0.813)

**PKH**	**PCa**	**PSA<10**	**PI-RADS ≥3**	723	100 (13.8%)	623 (86.2%)	0.813 (0.765-0.858)	0.760 (0.668-0.833)	0.790 (0.756-0.820)	0.367 (0.304-0.435)	0.953 (0.932-0.969)
			**PI-RADS ≥4**				0.813 (0.765-0.858)	0.570 (0.472-0.663)	0.886 (0.859-0.909)	0.445 (0.362-0.532)	0.928 (0.904-0.946)
		**10≤PSA≤20**	**PI-RADS ≥3**	409	97 (23.7%)	312 (76.3%)	0.825 (0.774-0.876)	0.835 (0.749-0.896)	0.718 (0.666-0.765)	0.479 (0.405-0.554)	0.933 (0.894-0.959)
			**PI-RADS ≥4**				0.825 (0.774-0.876)	0.691 (0.593-0.774)	0.833 (0.788-0.871)	0.563 (0.473-0.649)	0.897 (0.856-0.927)
		**PSA>20**	**PI-RADS ≥3**	346	235 (67.9%)	111 (32.1%)	0.817 (0.764-0.866)	0.979 (0.951-0.991)	0.495 (0.404-0.587)	0.804 (0.754-0.846)	0.917 (0.819-0.964)
			**PI-RADS ≥4**				0.817 (0.764-0.866)	0.940 (0.902-0.964)	0.604 (0.511-0.690)	0.834 (0.784-0.874)	0.827 (0.731-0.894)
	**csPCa**	**PSA<10**	**PI-RADS ≥3**	723	72 (10.0%)	651 (90.0%)	0.843 (0.788-0.889)	0.833 (0.731-0.902)	0.774 (0.741-0.805)	0.290 (0.232-0.355)	0.977 (0.960-0.987)
			**PI-RADS ≥4**				0.843 (0.788-0.889)	0.667 (0.552-0.765)	0.877 (0.850-0.900)	0.375 (0.296-0.461)	0.960 (0.941-0.973)
		**10≤PSA≤20**	**PI-RADS ≥3**	409	81 (19.8%)	328 (80.2%)	0.851 (0.801-0.898)	0.877 (0.787-0.932)	0.701 (0.650-0.748)	0.420 (0.348-0.495)	0.958 (0.925-0.977)
			**PI-RADS ≥4**				0.851 (0.801-0.898)	0.765 (0.662-0.844)	0.826 (0.781-0.863)	0.521 (0.432-0.609)	0.934 (0.900-0.958)
		**PSA>20**	**PI-RADS ≥3**	346	195 (56.4%)	151 (43.6%)	0.734 (0.681-0.783)	0.979 (0.948-0.992)	0.371 (0.298-0.450)	0.668 (0.611-0.720)	0.933 (0.841-0.974)
			**PI-RADS ≥4**				0.734 (0.681-0.783)	0.944 (0.902-0.968)	0.464 (0.386-0.543)	0.694 (0.636-0.747)	0.864 (0.773-0.922)
**REAC**	**PCa**	**PSA<10**	**PI-RADS ≥3**	514	140 (27.2%)	374 (72.8%)	0.840 (0.800-0.876)	0.886 (0.822-0.928)	0.631 (0.581-0.678)	0.473 (0.414-0.534)	0.937 (0.899-0.961)
			**PI-RADS ≥4**				0.840 (0.800-0.876)	0.579 (0.496-0.657)	0.928 (0.897-0.950)	0.750 (0.661-0.822)	0.855 (0.817-0.886)
		**10≤PSA≤20**	**PI-RADS ≥3**	321	104 (32.4%)	217 (67.6%)	0.834 (0.783-0.881)	0.904 (0.832-0.947)	0.548 (0.482-0.613)	0.490 (0.420-0.560)	0.922 (0.863-0.957)
			**PI-RADS ≥4**				0.834 (0.783-0.881)	0.654 (0.558-0.738)	0.908 (0.862-0.940)	0.773 (0.675-0.848)	0.845 (0.794-0.886)
		**PSA>20**	**PI-RADS ≥3**	338	242 (71.6%)	96 (28.4%)	0.893 (0.853-0.926)	0.959 (0.926-0.977)	0.458 (0.362-0.558)	0.817 (0.768-0.858)	0.815 (0.692-0.896)
			**PI-RADS ≥4**				0.893 (0.853-0.926)	0.843 (0.792-0.883)	0.802 (0.711-0.869)	0.915 (0.871-0.945)	0.670 (0.579-0.749)

	**csPCa**	**PSA<10**	**PI-RADS ≥3**	514	85 (16.5%)	429 (83.5%)	0.875 0.909)	(0.834-	0.953 (0.885-0.982)	0.578 (0.531-0.624)	0.309 (0.256-0.368)	0.984 (0.960-0.994)
			**PI-RADS ≥4**				0.875 0.909)	(0.834-	0.706 (0.602-0.792)	0.888 (0.855-0.915)	0.556 (0.462-0.646)	0.938 (0.911-0.958)
		**10≤PSA≤20**	**PI-RADS ≥3**	321	76 (23.7%)	245 (76.3%)	0.871 0.913)	(0.822-	0.961 (0.890-0.986)	0.514 (0.452-0.576)	0.380 (0.315-0.451)	0.977 (0.934-0.992)
			**PI-RADS ≥4**				0.871 0.913)	(0.822-	0.750 (0.642-0.834)	0.873 (0.826-0.909)	0.648 (0.544-0.739)	0.918 (0.876-0.947)
		**PSA>20**	**PI-RADS ≥3**	338	233 (68.9%)	105 (31.1%)	0.873 0.912)	(0.832-	0.966 (0.934-0.983)	0.438 (0.347-0.534)	0.792 (0.741-0.835)	0.852 (0.734-0.923)
			**PI-RADS ≥4**				0.873 0.912)	(0.832-	0.845 (0.794-0.886)	0.752 (0.662-0.825)	0.883 (0.835-0.919)	0.687 (0.597-0.765)
**SUM**	**PCa**	**PSA<10**	**PI-RADS ⩾3**	5885	1836 (31.2%)	4049 (68.8%)	0.799 0.811)	(0.788-	0.842 (0.825-0.858)	0.587 (0.571-0.602)	0.480 (0.463-0.497)	0.891 (0.879-0.902)
			**PI-RADS ⩾4**				0.799 0.811)	(0.788-	0.656 (0.634-0.677)	0.846 (0.835-0.857)	0.659 (0.637-0.681)	0.844 (0.833-0.855)
		**10⩽PSA⩽20**	**PI-RADS ⩾3**	3705	1751 (47.3%)	1954 (52.7%)	0.832 0.844)	(0.818-	0.880 (0.864-0.894)	0.569 (0.547-0.591)	0.647 (0.627-0.666)	0.841 (0.820-0.860)
			**PI-RADS ⩾4**				0.832 0.844)	(0.818-	0.742 (0.721-0.762)	0.839 (0.822-0.854)	0.805 (0.785-0.823)	0.784 (0.766-0.801)
		**PSA>20**	**PI-RADS ⩾3**	4064	3179 (78.2%)	885 (21.8%)	0.872 0.885)	(0.858-	0.959 (0.952-0.966)	0.478 (0.445-0.511)	0.868 (0.857-0.879)	0.766 (0.729-0.800)
			**PI-RADS ⩾4**				0.872 0.885)	(0.858-	0.895 (0.884-0.905)	0.748 (0.718-0.776)	0.927 (0.918-0.936)	0.665 (0.635-0.693)
	**csPCa**	**PSA<10**	**PI-RADS ⩾3**	5885	1416 (24.1%)	4469 (75.9%)	0.818 0.830)	(0.806-	0.879 (0.860-0.895)	0.558 (0.543-0.572)	0.386 (0.370-0.403)	0.935 (0.925-0.944)
			**PI-RADS ⩾4**				0.818 0.830)	(0.806-	0.723 (0.699-0.746)	0.821 (0.809-0.832)	0.561 (0.538-0.583)	0.903 (0.894-0.912)
		**10⩽PSA⩽20**	**PI-RADS ⩾3**	3705	1540 (41.6%)	2165 (58.4%)	0.838 0.851)	(0.826-	0.905 (0.889-0.918)	0.543 (0.522-0.564)	0.585 (0.565-0.604)	0.889 (0.871-0.905)
			**PI-RADS ⩾4**				0.838 0.851)	(0.826-	0.785 (0.764-0.805)	0.813 (0.796-0.829)	0.749 (0.727-0.770)	0.842 (0.825-0.857)
		**PSA>20**	**PI-RADS ⩾3**	4064	3055 (75.2%)	1009 (24.8%)	0.852 0.866)	(0.837-	0.966 (0.959-0.972)	0.445 (0.415-0.476)	0.841 (0.828-0.852)	0.813 (0.779-0.844)

	**PI-RADS ⩾4**	0.852 (0.837-0.866)	0.905 (0.894-0.915)	0.700 (0.671-0.727)	0.901 (0.890-0.911)	0.709 (0.680-0.736)

Table S7 Subgroup analysis of PI-RADS in predicting PCa and csPCa by MRI report time（<2020 and ≥2020）

Centers	Outcome	MRI report time	Cutoff	No.	Positive	Negative	AUC	SEN	SPE	PPV	NPV
**SCUH**	**PCa**	**<2020**	**PI-RADS ≥3**	1802	1023 (56.8%)	779 (43.2%)	0.769 (0.746-0.790)	0.915 (0.896-0.931)	0.418 (0.384-0.453)	0.674 (0.649-0.698)	0.789 (0.747-0.826)
			**PI-RADS ≥4**				0.769 (0.746-0.790)	0.796 (0.770-0.819)	0.685 (0.652-0.717)	0.769 (0.742-0.793)	0.719 (0.685-0.750)
		**≥2020**	**PI-RADS ≥3**	4078	2235 (54.8%)	1843 (45.2%)	0.794 (0.780-0.807)	0.929 (0.918-0.939)	0.353 (0.331-0.375)	0.635 (0.619-0.651)	0.804 (0.776-0.830)
			**PI-RADS ≥4**				0.794 (0.780-0.807)	0.800 (0.783-0.816)	0.660 (0.638-0.681)	0.740 (0.723-0.757)	0.731 (0.709-0.752)
	**csPCa**	**<2020**	**PI-RADS ≥3**	1802	942 (52.3%)	860 (47.7%)	0.768 (0.747-0.788)	0.920 (0.901-0.936)	0.393 (0.361-0.426)	0.624 (0.598-0.649)	0.818 (0.778-0.853)
			**PI-RADS ≥4**				0.768 (0.747-0.788)	0.818 (0.793-0.842)	0.665 (0.633-0.696)	0.728 (0.700-0.754)	0.770 (0.738-0.799)
		**≥2020**	**PI-RADS ≥3**	4078	2069 (50.7%)	2009 (49.3%)	0.806 (0.793-0.819)	0.943 (0.932-0.952)	0.343 (0.323-0.365)	0.597 (0.580-0.613)	0.854 (0.828-0.877)
			**PI-RADS ≥4**				0.806 (0.793-0.819)	0.826 (0.809-0.841)	0.648 (0.627-0.669)	0.707 (0.689-0.725)	0.783 (0.762-0.802)
**NJUH**	**PCa**	**<2020**	**PI-RADS ≥3**	2031	1091 (53.7%)	940 (46.3%)	0.858 (0.842-0.874)	0.918 (0.900-0.932)	0.496 (0.464-0.528)	0.679 (0.654-0.702)	0.838 (0.805-0.866)
			**PI-RADS ≥4**				0.858 (0.842-0.874)	0.808 (0.784-0.831)	0.823 (0.798-0.846)	0.842 (0.818-0.862)	0.787 (0.761-0.812)
		**≥2020**	**PI-RADS ≥3**	3111	1832 (58.9%)	1279 (41.1%)	0.864 (0.852-0.876)	0.939 (0.928-0.949)	0.463 (0.436-0.490)	0.715 (0.696-0.732)	0.842 (0.813-0.867)
			**PI-RADS ≥4**				0.864 (0.852-0.876)	0.855 (0.838-0.871)	0.762 (0.737-0.784)	0.837 (0.820-0.853)	0.786 (0.762-0.808)
	**csPCa**	**<2020**	**PI-RADS ≥3**	2031	944 (46.5%)	1087 (53.5%)	0.870 (0.854-0.886)	0.944 (0.927-0.957)	0.463 (0.433-0.492)	0.604 (0.579-0.629)	0.905 (0.877-0.926)
			**PI-RADS ≥4**				0.870 (0.854-0.886)	0.860 (0.837-0.881)	0.783 (0.757-0.806)	0.775 (0.749-0.799)	0.866 (0.843-0.886)
		**≥2020**	**PI-RADS ≥3**	3111	1673 (53.8%)	1438 (46.2%)	0.873 (0.861-0.884)	0.956 (0.945-0.965)	0.437 (0.412-0.463)	0.664 (0.645-0.683)	0.895 (0.870-0.915)
			**PI-RADS ≥4**				0.873 (0.861-0.884)	0.889 (0.873-0.904)	0.733 (0.709-0.755)	0.795 (0.776-0.813)	0.851 (0.830-0.869)
**TJUH**	**PCa**	**<2020**	**PI-RADS ≥3**	5738	3158 (55.0%)	2580 (45.0%)	0.825 (0.814-0.836)	0.900 (0.889-0.910)	0.531 (0.512-0.550)	0.701 (0.687-0.715)	0.813 (0.794-0.831)

			PI-RADS ≥4				0.825 (0.814-0.836)	0.786 (0.771-0.800)	0.794 (0.778-0.809)	0.824 (0.810-0.837)	0.752 (0.735-0.768)
		≥2020	PI-RADS ≥3	12040	6530 (54.2%)	5510 (45.8%)	0.828 (0.821-0.835)	0.913 (0.906-0.919)	0.458 (0.445-0.472)	0.666 (0.657-0.676)	0.816 (0.802-0.829)
			PI-RADS ≥4				0.828 (0.821-0.835)	0.797 (0.787-0.807)	0.758 (0.746-0.769)	0.796 (0.786-0.806)	0.759 (0.748-0.770)
	csPCa	<2020	PI-RADS ≥3	5738	2830 (49.3%)	2908 (50.7%)	0.832 (0.821-0.842)	0.919 (0.908-0.928)	0.500 (0.482-0.519)	0.642 (0.627-0.656)	0.864 (0.846-0.879)
			PI-RADS ≥4				0.832 (0.821-0.842)	0.821 (0.807-0.835)	0.763 (0.748-0.779)	0.772 (0.756-0.786)	0.814 (0.799-0.829)
		≥2020	PI-RADS ≥3	12040	5965 (49.5%)	6075 (50.5%)	0.838 (0.831-0.845)	0.930 (0.924-0.936)	0.441 (0.429-0.454)	0.620 (0.610-0.630)	0.866 (0.853-0.877)
			PI-RADS ≥4				0.838 (0.831-0.845)	0.828 (0.818-0.837)	0.736 (0.725-0.747)	0.755 (0.744-0.765)	0.813 (0.803-0.823)
**ZJH**	**PCa**	**<2020**	**PI-RADS ≥3**	367	197 (53.7%)	170 (46.3%)	0.813 (0.767-0.854)	0.827 (0.769-0.874)	0.624 (0.549-0.693)	0.718 (0.656-0.773)	0.757 (0.680-0.821)
			**PI-RADS ≥4**				0.813 (0.767-0.854)	0.624 (0.555-0.689)	0.900 (0.846-0.937)	0.879 (0.814-0.923)	0.674 (0.611-0.732)
		**≥2020**	**PI-RADS ≥3**	932	499 (53.5%)	433 (46.5%)	0.838 (0.813-0.864)	0.928 (0.902-0.947)	0.406 (0.361-0.453)	0.643 (0.607-0.677)	0.830 (0.774-0.875)
			**PI-RADS ≥4**				0.838 (0.813-0.864)	0.790 (0.752-0.823)	0.778 (0.737-0.815)	0.804 (0.767-0.837)	0.762 (0.721-0.800)
	**csPCa**	**<2020**	**PI-RADS ≥3**	367	169 (46.0%)	198 (54.0%)	0.834 (0.791-0.872)	0.876 (0.818-0.917)	0.601 (0.532-0.667)	0.652 (0.588-0.711)	0.850 (0.782-0.900)
			**PI-RADS ≥4**				0.834 (0.791-0.872)	0.680 (0.607-0.746)	0.874 (0.820-0.913)	0.821 (0.750-0.876)	0.762 (0.703-0.813)
		**≥2020**	**PI-RADS ≥3**	932	441 (47.3%)	491 (52.7%)	0.851 (0.826-0.876)	0.943 (0.918-0.961)	0.381 (0.339-0.425)	0.578 (0.541-0.613)	0.882 (0.832-0.919)
			**PI-RADS ≥4**				0.851 (0.826-0.876)	0.839 (0.802-0.870)	0.756 (0.716-0.792)	0.755 (0.715-0.791)	0.839 (0.802-0.871)
**ZJCH**	**PCa**	**<2020**	**PI-RADS ≥3**	154	99 (64.3%)	55 (35.7%)	0.785 (0.709-0.853)	0.838 (0.753-0.898)	0.545 (0.415-0.670)	0.769 (0.681-0.838)	0.652 (0.508-0.773)
			**PI-RADS ≥4**				0.785 (0.709-0.853)	0.697 (0.600-0.779)	0.855 (0.738-0.924)	0.896 (0.808-0.946)	0.610 (0.499-0.712)
		**≥2020**	**PI-RADS ≥3**	741	483 (65.2%)	258 (34.8%)	0.794 (0.759-0.827)	0.845 (0.810-0.874)	0.581 (0.520-0.640)	0.791 (0.754-0.824)	0.667 (0.603-0.725)
			**PI-RADS ≥4**				0.794 (0.759-0.827)	0.756 (0.715-0.792)	0.775 (0.720-0.822)	0.863 (0.827-0.892)	0.629 (0.575-0.680)
	**csPCa**	**<2020**	**PI-RADS ≥3**	154	85 (55.2%)	69 (44.8%)	0.804 (0.734-0.868)	0.871 (0.783-0.926)	0.507 (0.392-0.622)	0.685 (0.593-0.765)	0.761 (0.621-0.861)

			**PI-RADS ≥4**				0.804 (0.734-0.868)	0.741 (0.639-0.822)	0.797 (0.688-0.875)	0.818 (0.718-0.888)	0.714 (0.605-0.803)
		**≥2020**	**PI-RADS ≥3**	741	430 (58.0%)	311 (42.0%)	0.771 (0.739-0.802)	0.858 (0.822-0.888)	0.527 (0.472-0.582)	0.715 (0.675-0.752)	0.729 (0.667-0.783)
			**PI-RADS ≥4**				0.771 (0.739-0.802)	0.765 (0.723-0.803)	0.698 (0.645-0.746)	0.778 (0.736-0.815)	0.682 (0.629-0.731)
**SUM**	**PCa**	**<2020**	**PI-RADS ≥3**	5738	3158 (55.0%)	2580 (45.0%)	0.825 (0.814-0.836)	0.900 (0.889-0.910)	0.531 (0.512-0.550)	0.701 (0.687-0.715)	0.813 (0.794-0.831)
			**PI-RADS ≥4**				0.825 (0.814-0.836)	0.786 (0.771-0.800)	0.794 (0.778-0.809)	0.824 (0.810-0.837)	0.752 (0.735-0.768)
		**≥2020**	**PI-RADS ≥3**	12040	6530 (54.2%)	5510 (45.8%)	0.828 (0.821-0.835)	0.913 (0.906-0.919)	0.458 (0.445-0.472)	0.666 (0.657-0.676)	0.816 (0.802-0.829)
			**PI-RADS ≥4**				0.828 (0.821-0.835)	0.797 (0.787-0.807)	0.758 (0.746-0.769)	0.796 (0.786-0.806)	0.759 (0.748-0.770)
	**csPCa**	**<2020**	**PI-RADS ≥3**	5738	2830 (49.3%)	2908 (50.7%)	0.832 (0.821-0.842)	0.919 (0.908-0.928)	0.500 (0.482-0.519)	0.642 (0.627-0.656)	0.864 (0.846-0.879)
			**PI-RADS ≥4**				0.832 (0.821-0.842)	0.821 (0.807-0.835)	0.763 (0.748-0.779)	0.772 (0.756-0.786)	0.814 (0.799-0.829)
		**≥2020**	**PI-RADS ≥3**	12040	5965 (49.5%)	6075 (50.5%)	0.838 (0.831-0.845)	0.930 (0.924-0.936)	0.441 (0.429-0.454)	0.620 (0.610-0.630)	0.866 (0.853-0.877)
			**PI-RADS ≥4**				0.838 (0.831-0.845)	0.828 (0.818-0.837)	0.736 (0.725-0.747)	0.755 (0.744-0.765)	0.813 (0.803-0.823)

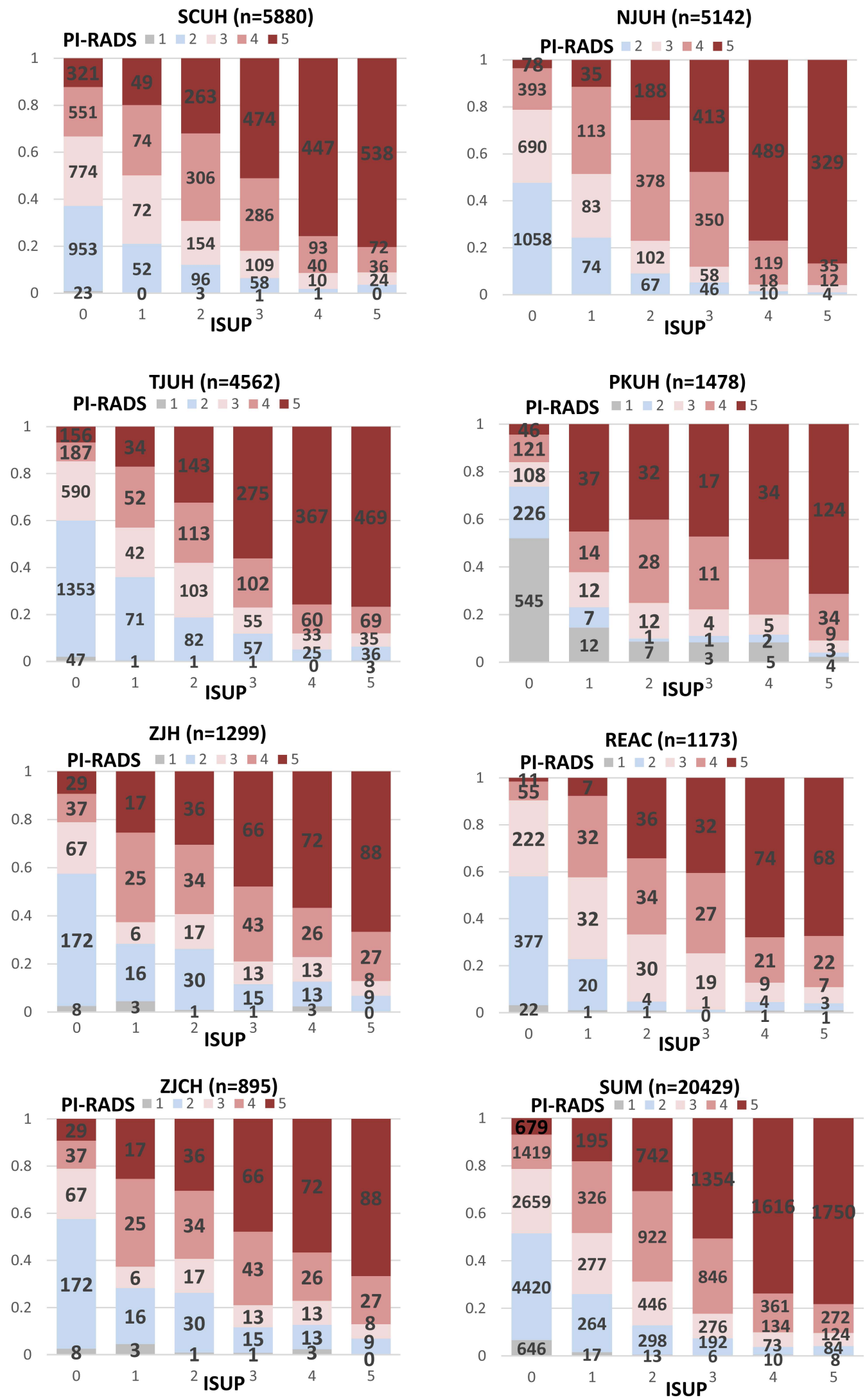
SCUH (n=5880)
NJUH (n=5142)
TJUH (n=4562)
PKUH (n=1478)
ZJH (n=1299)
REAC (n=1173)
ZJCH (n=895)
SUM (n=20429)
PI-RADS 1 2 3 4 5
ISUP

Figure S1 The distribution of ISUP grade stratified by PI-RADS each score was plotted to illustrate relationship of PI-RADS score with ISUP grade. Notes: ISUP, International Society of Urological Pathology; SCUH, the First Affiliated Hospital of Soochow University; NJUH, the First Affiliated Hospital of Nanjing Medical University; TJUH, Tongji Hospital of Tongji Medical college; PKUH, Peking University First Hospital; ZJH, Zhangjiagang Hospital of Suzhou University; ZJCH, Zhejiang Cancer Hospital; REAC, a cross-district re-alliancing center.

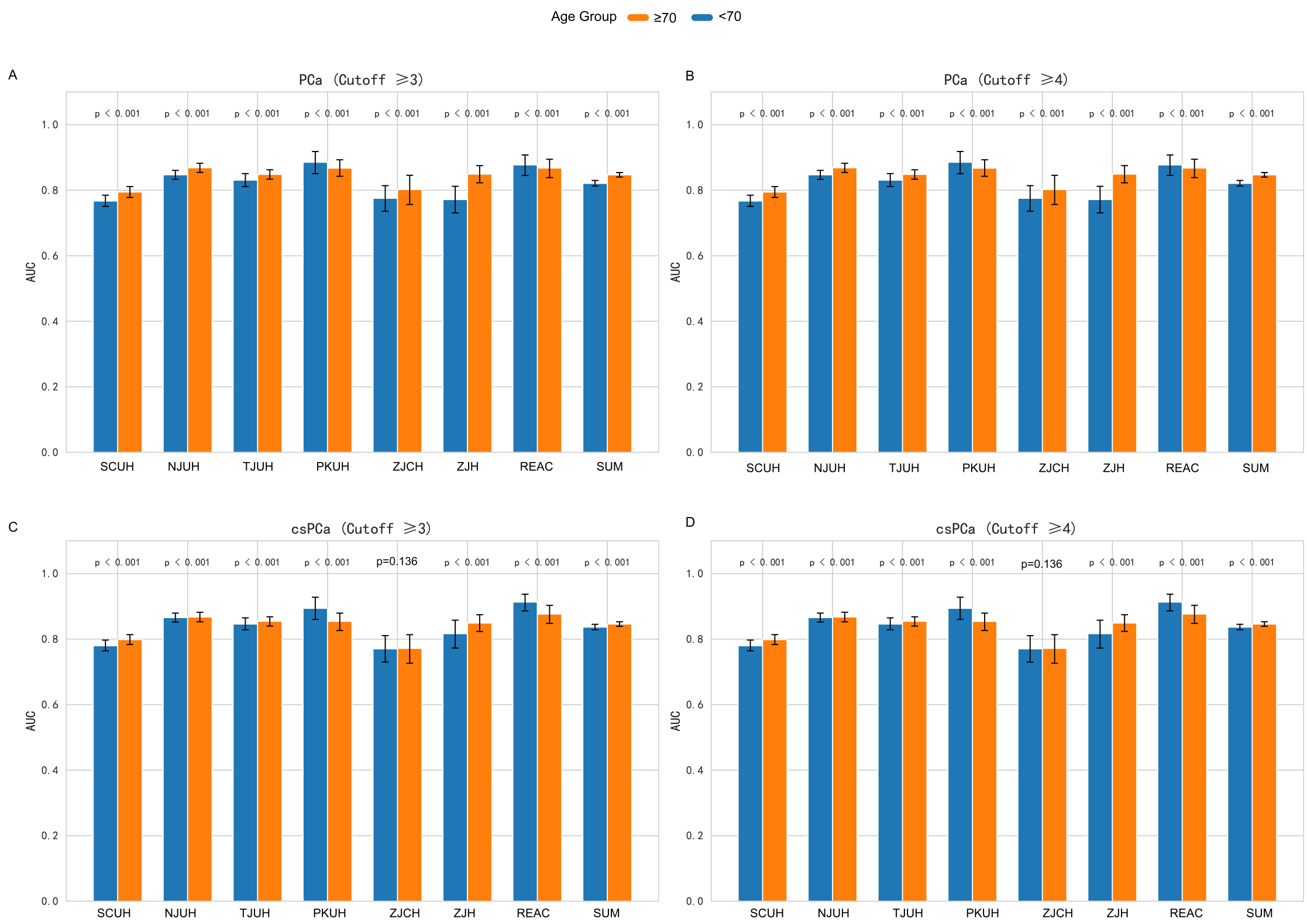

Figure S2. Diagnostic performance comparison, as measured by the area under the receiver operating characteristic curve (AUC), across different institutions for detecting prostate cancer (PCa) (A and B) and clinically significant prostate cancer (csPCa) (C and D) in patient subgroups stratified by age (<70 years vs. ≥70 years). Performance is shown for PI-RADS cutoff scores of both ≥3 and ≥4. The analysis is performed across multiple centers (SCUH, NJUH, TJUH, PKUH, ZJCH, ZJH, REAC) and includes a summary (SUM) of all centers. The *p* values above the bars represent the statistical significance of the difference in AUC values between the two groups for each center, calculated using appropriate statistical tests. *Notes: PCa = prostate cancer; csPCa = clinically significant prostate cancer; AUC = area under the receiver operating characteristic curve. SCUH, the First Affiliated Hospital of Soochow University; NJUH, the First Affiliated*

Hospital of Nanjing Medical University; TJUH, Tongji Hospital of Tongji Medical college; PKUH, Peking University First Hospital; ZJH, Zhangjiagang Hospital of Suzhou University; ZJCH, Zhejiang Cancer Hospital; REAC, a cross-district re-alliancing center.

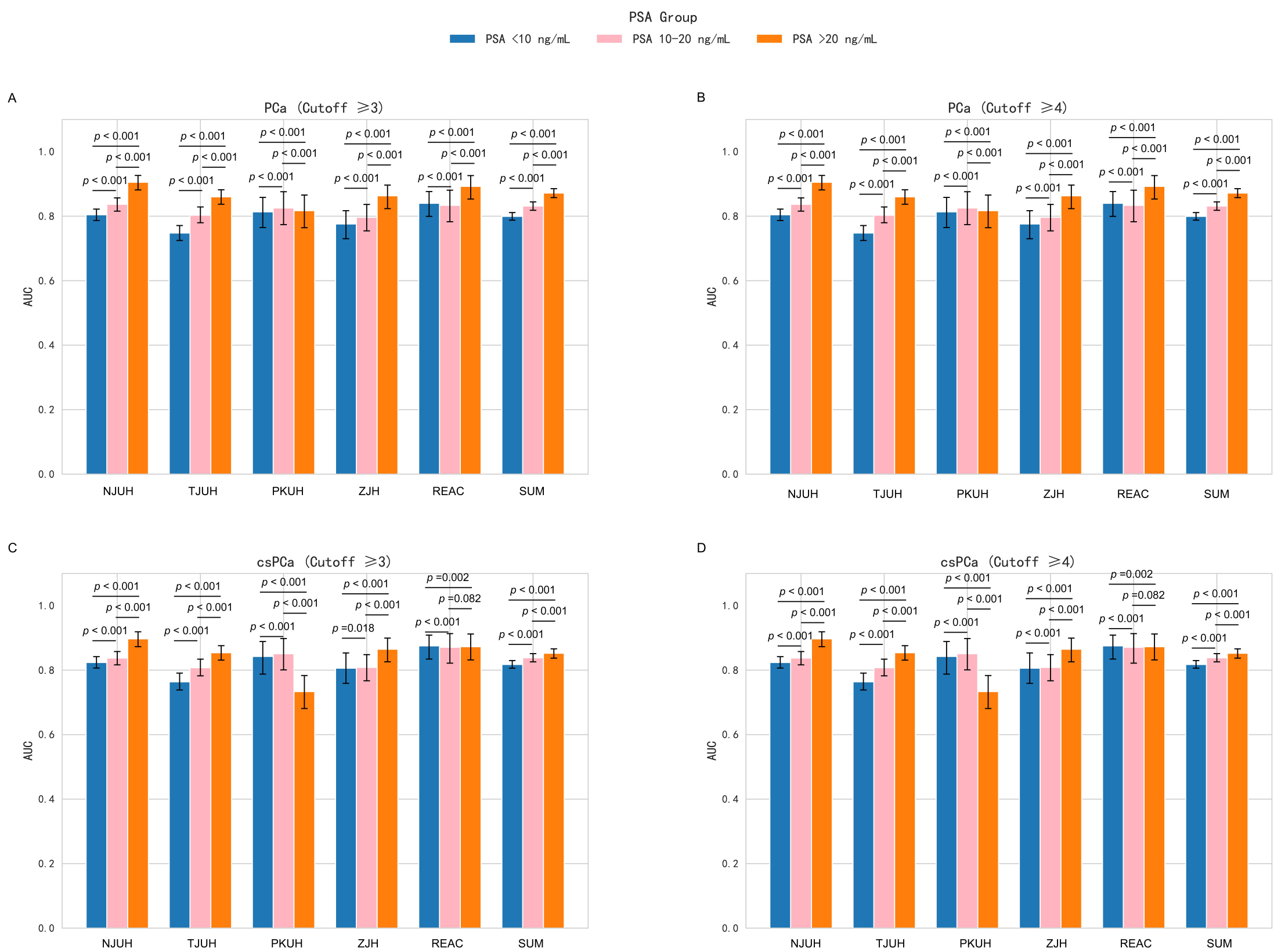

Figure S3. Diagnostic performance (AUC) by PSA subgroup across centers for detection of prostate cancer (PCa) and clinically significant PCa (csPCa) at PI-RADS cutoffs ≥3 (A, C) and ≥4 (B, D). Each panel shows AUC values for three PSA groups (<10 ng/mL, 10–20 ng/mL, and >20 ng/mL) across the indicated centers and the summary results (SUM). Short black lines indicate within-center pairwise AUC comparisons between PSA groups; *p* values denote statistical significance from

DeLong's test. A trend of increasing AUC values with rising PSA levels is observed in most centers. For PCa detection, at both PI-RADS cutoffs, the >20 ng/mL PSA group generally shows higher AUC values compared to the <10 ng/mL group in all centers. Similarly, for csPCa detection, significant differences between PSA groups are noted in some centers. However, REAC had no significant differences in AUC values among the PSA subgroups. The SUM further consolidates the relationship between PSA levels and the diagnostic performance of PI-RADS. *Notes: PCa = prostate cancer; csPCa = clinically significant prostate cancer; AUC = area under the receiver operating characteristic curve; SCUH, the First Affiliated Hospital of Soochow University; NJUH, the First Affiliated Hospital of Nanjing Medical University; TJUH, Tongji Hospital of Tongji Medical college; PKUH, Peking University First Hospital; ZJH, Zhangjiagang Hospital of Suzhou University; ZJCH, Zhejiang Cancer Hospital; REAC, a cross-district re-alliancing center.*

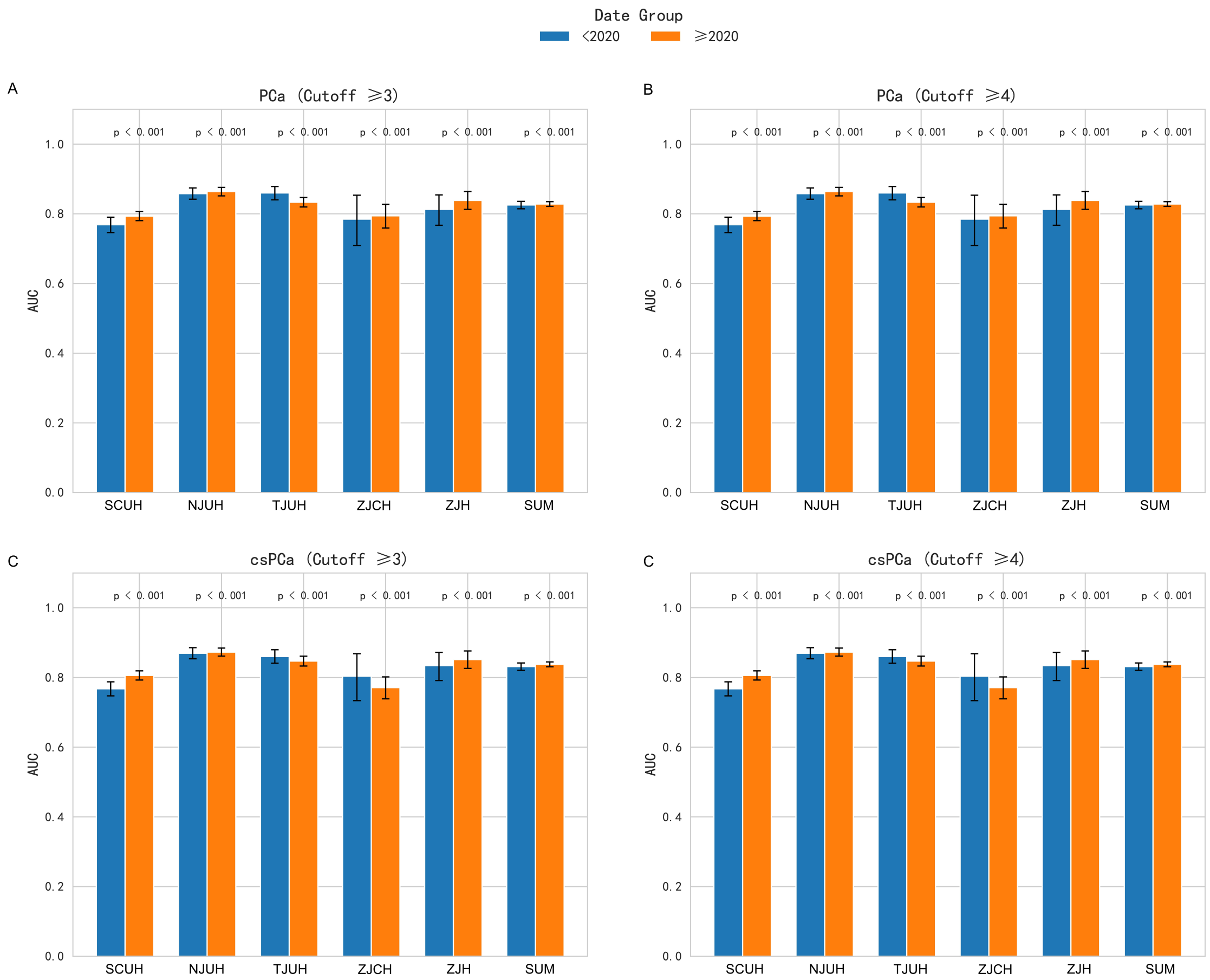

Figure S4. Diagnostic performance (AUC) of PI-RADS for detection of prostate cancer (PCa) and clinically significant prostate cancer (csPCa) stratified by MRI report time (<2020 vs. ≥2020) across participating centers, along with a summary (SUM) of the results. The analysis is conducted using two diagnostic thresholds (cutoff ≥3 and cutoff ≥4). Each bar represents the mean AUC with error bars indicating the 95% confidence interval (CI). Blue bars represent cases scanned before 2020 (<2020), and orange bars represent cases scanned in or after 2020 (≥2020).

The *p* values indicate statistically significant differences between the two groups for the same center. *Notes: The PKUH center was excluded from this PI-RADS version subgroup analysis (<2020 vs. ≥2020) because exact examination dates at the individual case level were unavailable, precluding reliable assignment of cases to the appropriate PI-RADS version era. This exclusion was applied proactively to ensure methodological rigor of the version comparison. PCa = prostate cancer; csPCa = clinically significant prostate cancer; AUC = area under the receiver operating characteristic curve.*
